# A novel transient receptor potential C3/C6 selective activator induces the cellular uptake of antisense oligonucleotides

**DOI:** 10.1093/nar/gkae245

**Published:** 2024-04-16

**Authors:** Hiroto Kohashi, Ryu Nagata, Yusuke Tamenori, Tomorrow Amatani, Yoshifumi Ueda, Yasuo Mori, Yuuya Kasahara, Satoshi Obika, Masahito Shimojo

**Affiliations:** Graduate School of Pharmaceutical Sciences, Osaka University, Osaka 565-0871, Japan; Graduate School of Pharmaceutical Sciences, Osaka University, Osaka 565-0871, Japan; School of Pharmaceutical Sciences, Osaka University, Osaka 565-0871, Japan; Graduate School of Engineering, Kyoto University, Kyoto 615-8510, Japan; Graduate School of Engineering, Kyoto University, Kyoto 615-8510, Japan; Graduate School of Engineering, Kyoto University, Kyoto 615-8510, Japan; National Institutes of Biomedical Innovation, Health and Nutrition, Osaka 567-0085, Japan; Graduate School of Pharmaceutical Sciences, Osaka University, Osaka 565-0871, Japan; Institute for Open and Transdisciplinary Research Initiatives (OTRI), Osaka University, Osaka 565-0871, Japan; Graduate School of Pharmaceutical Sciences, Osaka University, Osaka 565-0871, Japan

## Abstract

Antisense oligonucleotide (ASO) therapy is a novel therapeutic approach in which ASO specifically binds target mRNA, resulting in mRNA degradation; however, cellular uptake of ASOs remains critically low, warranting improvement. Transient receptor potential canonical (TRPC) channels regulate Ca^2+^ influx and are activated upon stimulation by phospholipase C-generated diacylglycerol. Herein, we report that a novel TRPC3/C6/C7 activator, L687, can induce cellular ASO uptake. L687-induced ASO uptake was enhanced in a dose- and incubation-time-dependent manner. L687 enhanced the knockdown activity of various ASOs both *in vitro* and *in vivo*. Notably, suppression of TRPC3/C6 by specific siRNAs reduced ASO uptake in A549 cells. Application of BAPTA-AM, a Ca^2+^ chelator, and SKF96365, a TRPC3/C6 inhibitor, suppressed Ca^2+^ influx via TRPC3/C6, resulting in reduced ASO uptake, thereby suggesting that Ca^2+^ influx via TRPC3/C6 is critical for L687-mediated increased ASO uptake. L687 also induced dextran uptake, indicating that L687 increased endocytosis. Adding ASO to L687 resulted in endosome accumulation; however, the endosomal membrane disruptor UNC7938 facilitated endosomal escape and enhanced knockdown activity. We discovered a new function for TRPC activators regarding ASO trafficking in target cells. Our findings provide an opportunity to formulate an innovative drug delivery system for the therapeutic development of ASO.

## Graphical Abstract

**Graphical Abstract:**
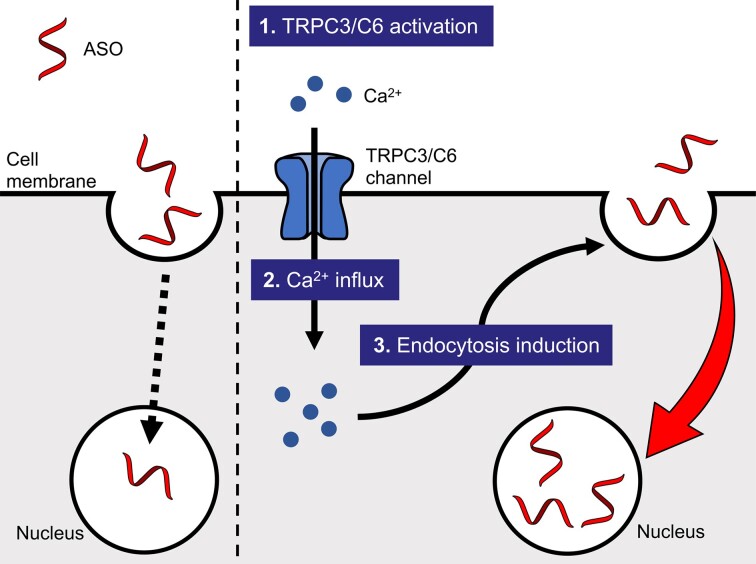

## Introduction

Oligonucleotide therapeutics, such as antisense oligonucleotides (ASOs) and small interfering RNAs (siRNAs), have been shown to directly regulate protein expression (1), which is not feasible with small-molecule drugs and antibodies. Given their unique mechanisms in controlling the production of disease-related proteins via the regulation of gene expression (2), oligonucleotide therapeutics could be applied to various genetic diseases (3). Of the 18 currently approved oligonucleotide therapeutics, 15 act on RNA (4), and RNA-targeted oligonucleotides have been actively studied. Single-stranded oligonucleotides with artificial nucleic acids are taken up into cells by endocytosis and specifically bind to target mRNA, leading to various downstream effects on the regulation of disease-related gene expression (5) via RNA cleavage or splicing changes (6). Chemical modifications have also been explored, such as introducing artificial nucleic acids to enhance affinity to target sequences, stability, and cellular uptake (7). However, low intracellular uptake owing to the polyanionic and hydrophilic nature of oligonucleotides remains a major challenge (8). In addition, systemically administered oligonucleotides are predominantly distributed in specific organs, such as the liver and kidney (9). The selective delivery of oligonucleotides to target cells and their emerging activities remain major concerns that need to be resolved, and their versatility is currently restricted. The development of delivery systems has improved the safety and efficacy of ASO therapy (10). Importantly, improving oligonucleotide incorporation into target cells using specific strategies could, at least partially, aid in overcoming challenges that hinder ASO delivery to target organs following systemic administration.

To address the low intracellular translocation of ASOs, various oligonucleotide delivery systems have been developed, including lipid nanoparticle formulations (LNP) (11), DNA origami platforms (12), nano drug delivery systems (13) and ligand conjugation systems, such as cell membrane-permeable peptides (CPP) (14), N-acetylgalactosamine (GalNAc) (15), aptamers (16) and antibodies (AOC) (17). A transfection method, Ca^2+^-enrichment of medium (CEM), previously developed by our group, was found to improve the uptake of ASO and siRNA by enhancing the Ca^2+^ concentration in the medium (18,19). Although nanoparticles formed by serum and Ca^2+^ in the medium could potentially facilitate efficient oligonucleotide uptake, the underlying mechanism remains unknown (18). When using the CEM method, certain intracellular Ca^2+^ signalling may participate in oligonucleotide uptake. Although the CEM method is an alternative to the lipofection method for ASO transfection *in vitro*, its application *in vivo* and in clinical settings can be challenging.

Transient receptor potential (TRP) channels are non-selective cation channels located mainly in the plasma membrane to permeate inorganic cations such as Ca^2+^ and Na^+^. TRP channels are known to mobilize intracellular Ca^2+^ signalling that mediates important functions, for example, as sensors of extracellular and intracellular events, such as changes in temperature, pH, osmotic pressure, and mechanical stimulation (20). The transient receptor potential canonical (TRPC) subfamily is comprised of six functional members in humans: TRPC1 and TRPC3–7. TRPC3/C6/C7 forms a TRPC subgroup with high homology to each other, and are functionally characterized by activation sensitivity to diacylglycerol generated by phospholipase C (PLC) activation through hydrolysis of a membrane component phosphatidylinositol 4,5-bisphosphate (PI(4,5)P2), thereby implying that these channels participate in Ca^2+^ signalling (21–23). Importantly, TRPC3 mediates Ca^2+^ influx that drives secondary activation PLC and hydrolysis of PI(4,5)P_2_ and Ca^2+^ signalling (24). Several human TRPC channels are ubiquitously expressed in tissues and involved in a multitude of cellular responses (25). However, TRPC3/C6 channels are highly expressed in certain cancer cells, including lung, ovarian, and prostate cancer cells, and may exert specific functions that are yet to be comprehensively elucidated (26,27). Similar to CEM, which suggests a link between Ca^2+^ and ASO uptake, we focused on Ca^2+^-permeable TRP channels and their activators.

TRPC3/C6/C7 activators, capable of opening TRPC3/C6/C7 channels expressed on certain cell membranes, can introduce Ca^2+^ from the extracellular medium into the cytoplasm. Accordingly, we hypothesised that Ca^2+^ influx into cells mediated by the novel TRPC3/C6/C7 selective activator, L687, developed by our group, would improve intracellular ASO translocation. Moreover, the amount of ASO transferred into cells was assessed based on the hypothesis that L687 promotes cellular ASO uptake by inducing Ca^2+^ influx via TRPC3/C6/C7 channels. We also analysed the effect of L687 on ASO activity in TRPC3/C6/C7-expressing cancer cells and in a mouse xenograft model of cancer with transplanted cells. Based on our findings, we report that L687 could improve the intracellular uptake of ASO.

## Materials and methods

### Biological resources

All cell lines were obtained from the Japanese Collection of Research Bioresources Cell Bank (JCRB), the National Institutes of Biomedical Innovation, Health and Nutrition (Osaka, Japan), and the American Type Culture Collection (ATCC, VA, USA). The following cell lines were used: A549 (JCRB0076), NCI-N417 (CRL-5809), NCI-H146 (HTB-173), NCI-H209 (HTB-173), SK-OV-3 (HTB-77), HEK293 (CRL-1573) and 22Rv1 (CRL-2505). Briefly, cells were grown in appropriate media, such as DMEM (low-glucose) with l-glutamine and phenol red (#041-29775; FUJIFILM Wako Pure Chemical Co., Osaka, Japan) or RPMI-1640 medium (#187-02705; FUJIFILM Wako Pure Chemical Co., Osaka, Japan) containing 10% fetal bovine serum (FBS) (#10270-106; Thermo Fisher Scientific, MA, USA). Cells were cultured at 37°C in a humidified incubator under 5% CO_2_.

### Ca^2+^ influx analysis

For Ca^2+^ measurements, HEK293 cells were co-transfected with recombinant plasmids and pEGFP-N1 (Takara Bio USA, CA, USA) as a transfection marker using the PolyFect Transfection Reagent (Qiagen, MD, USA). Plasmids carrying mouse TRPC1α, TRPC2, TRPC3, TRPC4β, TRPC5, TRPC6, TRPC7, TRPA1, TRPM2, rat TRPV1 and cDNA were constructed as described previously (28). Cells were plated on glass coverslips 48 h after transfection, and Ca^2+^ measurements were performed 3–12 h after plating. After incubation with 1 μM Fura-2 AM (Thermo Fischer Scientific, MA, USA) in DMEM containing 10% FBS at 37°C for 30 min, cells on coverslips were placed in a perfusion chamber on the microscope stage. Fluorescence images of cells were recorded and analysed using AQUACOSMOS (Hamamatsu Photonics, Shizuoka, Japan). Fura-2 AM fluorescence at an emission wavelength of 510 nm (bandwidth, 20 nm) was obtained by alternately exciting Fura-2 AM at 340 and 380 nm (bandwidth, 11 nm). Images with a 340:380 nm ratio were produced on a pixel-by-pixel basis. All the reagents dissolved in water or DMSO were diluted to their final concentrations in HEPES-buffered saline containing 107 mM NaCl, 6 mM KCl, 1.2 mM MgSO_4_, 2 mM CaCl_2_, 11.5 mM glucose, 20 mM HEPES, adjusted to pH 7.4, with NaOH. The reagents were applied to cells through chamber perfusion.

### Oligonucleotide synthesis

ASOs used in this study were synthesised by Aji Bio-Pharma (Osaka, Japan). All ASOs possessed phosphorothioate backbone linkages. Oligonucleotides were purified by high-performance liquid chromatography and confirmed by mass spectrometry. ASOs are gapmer ASOs comprising natural DNA and modified nucleic acids, LNA or AmNA. Sequences of oligonucleotides used in the current study are as follows: AmNA#26-Alexa647:5′-TGAacaaaataaTAC-3′ (upper case: AmNA, lower case: DNA); SRRM4_ASO:5′- GTGactgaagcctcCAT-3′ (upper case: AmNA, lower case: DNA); REST_ASO:5′-GACtgtctattgctGGG-3′ (upper case: LNA, lower case: DNA); MALAT1_ASO:5′-GAGttacttgccaACT-3′ (upper case: LNA, lower case: DNA); ERBB2_ASO (HER2-LNA):5′-ACGtgtctgtgttgtAGG-3′ (upper case: LNA, lower case: DNA).

### TRPC3/C6/C7 activators and other reagents

The following TRPC3/C6/C7 activators were employed in the current study: L687 (Supplementary Figure S1, WO/2022/118966) (29), Cannabidiol (CBD) (#Axon1234; Axon Medchem, VA, USA), and GSK1702934A (#6508; Tocris Bioscience, Bristol, UK). SKF96365 (#1147/10; R&D Systems, MN, USA) was used as the TRPC channel inhibitor. BAPTA-AM (#B035; DOJINDO, Kumamoto, Japan) was used as the Ca^2+^ chelator. EIPA (#14406; Funakoshi Co., Tokyo, Japan) and Cytochalasin D (#034-25881; FUJIFILM Wako Pure Chemical Co., Osaka, Japan) were used as the macropinocytosis inhibitors. Nifedipine (#141-05783; FUJIFILM Wako Pure Chemical Co., Osaka, Japan) was used as the L-type calcium channel blocker. UNC7938 (#AOB13597; AOBIOUS Inc., MA, USA) was used as the destabilizer of the endosomal membrane. All reagents were dissolved in anhydrous DMSO (#D12345; Thermo Fisher Scientific, MA, USA) and added to the medium at a final concentration of 0.3–1% (v/v).

### Reverse transcription-quantitative polymerase chain reaction (RT-qPCR)

Total RNA was extracted using the RNeasy Plus Micro Kit (Qiagen, Hilden, Germany) according to the manufacturer's instructions. Total RNA was quantified at 260 nm using a spectrophotometer (#DS-11; DeNovix, DE, USA) and verified to be of high quality. Total RNA (1 μg/16 μl) was transcribed at 42°C for 60 min using 4 μl of SuperScript IV VILO Kit (#11756050; Thermo Fischer Scientific, MA, USA). Amplification was performed using the StepOnePlus Real-Time PCR System (Thermo Fisher Scientific, MA, USA). For *SRRM4*, forward and reverse primers (10 pmol each) were used in 20 μl reaction mixtures. For *β-actin*, the master mix was prepared using TaqMan™ β-actin Detection Reagent (#401846; Thermo Fisher Scientific, MA, USA). Each master mix was prepared using the TaqMan Fast Advanced Master Mix (#4444964; Thermo Fisher Scientific, MA, USA). RT-qPCR was performed to analyse aliquots of cDNA (1/10 of the RT reaction volume). qPCR was performed with an initial activation at 95°C for 20 s, followed by 40 amplification cycles (95°C for 3 s and 60°C for 30 s). Fluorescence development was assayed once per amplification cycle as recommended by the manufacturer. Relative mRNA levels were analysed using the 2-^ΔΔ^Ct method, with all ΔCt values normalised to those of *GAPDH* or *β-actin*. All qPCR experiments were performed at least in triplicate, and mean ± standard deviation values were calculated. PCR products were analysed as single bands on agarose gels, and DNA sequencing was performed to confirm the products. The primer sequences used were as follows: *SRRM4* forward:5′-TGACAAAGACTTGACACCACC-3′; *SRRM4* reverse:5′-ACCTGCGTCGCTTGTGTTT-3′; and TaqMan probe:5′-FAM-AGGTCCTCATCCTATAGCCCATCGCCT-TAMRA-3.’ Primers and probes were synthesised by Thermo Fisher Scientific (Waltham, MA, USA). The following TaqMan probe kits were used: *GAPDH* (Hs99999905_m1), *REST* (Hs05028212_s1), *MALAT1* (Hs00273907_s1), *TRPC3* (Hs00162985_m1) and *TRPC6* (Hs03928990_g1). For *ERBB2* analysis, SK-OV-3 cells seeded in 96-well black plates (Corning, NY, USA) at a density of 0.5 × 10^4^ cells/well were cultured for 12 h and then treated with ASO (100 nM in DMSO) and/or indicated concentrations of L687 for 48 h at 37°C. To evaluate *ERBB2* expression levels, mRNA was prepared from cell extracts using the PureLink RNA Mini Kit (Thermo Fischer Scientific, MA, USA) and reverse-transcribed into cDNA using PrimeScript™ RT reagent Kit (Takara Bio, Shiga, Japan). Real-time qPCR was performed on a QuantStudio 7 Flex Real-Time PCR System (Thermo Fischer Scientific, MA, USA) using Power SYBR Green PCR Mix (Thermo Fischer Scientific, MA, USA).

### Flow cytometry

Cells (2.0–8.0 × 10^4^/well) were seeded with 900 μl/well medium in 12-well plates (#3815-012; IWAKI, Shizuoka, Japan). After 24 h, the reagents such as Alexa647-AmNA#26 or Dextran, Alexa Fluor™ 647; 10,000 MW, Anionic, Fixable (#D22914; Thermo Fischer Scientific, MA, USA) (in 100 μl of medium) were added to the wells. The cells were detached using Accutase™ (#AT104; Innovative Cell Technologies, CA, USA) or trypsin/EDTA (#35554-64; Nacalai Tesque; Shizuoka, Japan). Subsequently, cells were washed with phosphate-buffered saline (PBS) and collected by centrifugation. Cells in PBS (#166-23555; FUJIFILM Wako Pure Chemical Co., Osaka, Japan) were filtered through a 40-μm strainer (#HT-AMS-14002; Hi-tech, Tokyo, Japan), followed by treatment with a final concentration of 300 nM 4′,6-diamidino-2-phenylindole, dihydrochloride (DAPI) (#D1306; Thermo Fisher Scientific, MA, USA) in an ice bath for 5 min. Fluorescence intensity (DAPI and Alexa647) was measured using a MACSQuant Analyzer 10 Flow Cytometer (Miltenyi Biotec, Bergisch Gladbach, Germany) and ZE5 Cell Analyzer (Bio-Rad, California, USA). Fluorescence intensity was analysed only for DAPI-unstained live cells, and the amount of ASO in cells was evaluated by comparing the average fluorescence intensity.

### Immunofluorescence staining

Briefly, cells (4.0 × 10^4^/well) were seeded in 900 μl of medium on a 12-well plate (#3815-012; IWAKI, Shizuoka, Japan). Each reagent was added after 24 h. Forty-eight hours after adding the reagent, the medium was removed, and cells were washed twice with PBS. Lysosome staining was performed using LysoTracker^®^ Green DND-26 (#L7526; Thermo Fisher Scientific, MA, USA) for 1.5 h under light-shielded conditions. Hoechst staining (10 ng/μl) in PBS was performed using Hoechst33342 (#H3570; Thermo Fisher Scientific, MA, USA) for 10 min under light-shielded conditions. Subsequently, cells were washed twice with PBS. Images were obtained using a fluorescence microscope BZ-9000 (Keyence, Osaka, Japan) or CellVoyagerCV8000 (Yokogawa Electric, Tokyo, Japan). For experiments using N417 cells, cells were attached to glass slides using Smear Gell (#SG-01; GenoStaff, Tokyo, Japan). Subsequently, fixation was performed using 4% paraformaldehyde phosphate buffer (#09154-14; Nacalai Tesque, Kyoto, Japan) for 30 min, followed by membrane permeabilisation using 10× diluted 0.05%–tTBS (10×) (pH 7.4) (#12749-21; Nacalai Tesque, Kyoto, Japan) for 30 min, followed by blocking with Normal Goat Serum (10%) (#50062Z; Thermo Fischer Scientific, MA, USA) for 30 min. Incubation with Anti-EEA1 Antibody-Early Endosome Marker (#ab2900; Abcam, Cambridge, UK) at 1:100 dilution was performed at room temperature for 1 h followed by incubation with Goat anti-rabbit IgG (H + L) Highly Cross-Adsorbed Secondary Antibody, Alexa Fluor™ Plus 488 (Thermo Fischer Scientific: # A32731) at 1:1000 dilution was performed at room temperature for 1 h while the light was shielded. Finally, cell nuclei were stained using SlowFade™ Gold Antifade Mountant with DAPI (#S36939; Thermo Fischer Scientific, MA, USA), mounted on a cover glass, and examined under a fluorescence microscope BZ-X800 (Keyence, Osaka, Japan).

### siRNA transfection

siRNAs were transfected into cells (70–80% confluence) using Lipofectamine^®^3000 (#L3000015; Thermo Fischer Scientific, MA, USA) according to the manufacturer's instructions. Briefly, cells (4.0 × 10^4^/well) cultured on 12-well plates (#3815-012; IWAKI, Shizuoka, Japan) were incubated with 3 μl of Lipofectamine^®^3000 in 1000 μl medium with varying amounts of siRNAs, as shown in each figure. Cells were then cultured at 37°C in a humidified incubator with 5% CO_2_. The following siRNAs were used: Silencer™ Select Negative Control No. 2 siRNA (#4390846; Thermo Fischer Scientific, MA, USA), Stealth RNAi™ siRNA (#HSS110984; Thermo Fischer Scientific, MA, USA), and Stealth RNAi™ siRNA (#HSS110994; Thermo Fischer Scientific, MA, USA).

### Western blot analysis

Protein extraction was performed in RIPA buffer (#08714-04; Nacalai Tesque, Kyoto, Japan). The cells were washed with ice-cold PBS and lysed in RIPA buffer on ice to promote complete cell lysis Then, lysates were sonicated and centrifuged at 10,000 *× g* for 15 min to collect the protein-containing supernatants. Protein concentrations in supernatants were determined using a Pierce™ BCA Protein Assay Kit (#23225; Thermo Fischer Scientific, MA, USA). Subsequently, 50 μg of each sample protein lysate was dissolved in 50  μl of Laemmli Sample Buffer (#1610737; Bio-Rad, CA, USA) containing 5% 2-mercaptoethanol (#131-14572; FUJIFILM Wako Pure Chemical Co., Osaka, Japan), separated by 7.5% Mini-PROTEAN TGX (#4561021; Bio-Rad, CA, USA), and transferred to PVDF membranes (#1704156; Bio-Rad, CA, USA). Membranes were blocked with 3% skim milk powder (#31149-75; Nacalai Tesque, Kyoto, Japan) in 0.05%–tTBS (#12749-21; Nacalai Tesque, Kyoto, Japan). Incubation with primary polyclonal rabbit anti-TRPC3 antibody (#ACC-016; Alomone Labs, Jerusalem, Israel), primary polyclonal rabbit anti-TRPC6 antibody (#ACC-120; Alomone Labs, Jerusalem, Israel) at 1:2000 dilution and mouse monoclonal anti-GAPDH (#AM4300; Thermo Fisher Scientific, MA, USA) at 1:2000 dilution was performed at 4°C overnight. Following incubation with the primary antibody, membranes were washed three times in 1 × TBS with 0.05% Tween-20 for 10 min and incubated for 1  h at room temperature with horseradish peroxidase (HRP)-conjugated goat anti-rabbit IgG (#SA00001-2; ProteinTech Group, Inc., Chicago, IL, USA) and HRP-conjugated goat anti-mouse IgG (#SA00001-1; ProteinTech Group, Inc., Chicago, IL, USA), diluted 1:5000 in blocking solution. Membranes were washed three times for 10 min in 0.05%–tTBS and finally developed with Chemi-Lumi One (#07880; Nacalai Tesque, Kyoto, Japan) or Chemi-Lumi Super (#02230; Nacalai Tesque, Kyoto, Japan) according to the manufacturer's instructions. Images obtained from the iBright^TM^ CL1500 Imaging System (Thermo Fisher Scientific, MA, USA) were analysed using ImageJ software (National Institutes of Health, Bethesda, MD). Western blot analyses were performed in triplicate.

### Cell viability

SK-OV-3 cells were seeded in a 96-well black plate (#3603; Corning, NY, USA) at a density of 1.0 × 10^4^ cells/well and cultured for 24 h at 37°C. Subsequently, cells were treated with ASO (100 nM in DMSO) and/or indicated concentrations of L687 for 48 h. To assess the number of cells in each well, cell nuclei were stained with Hoechst 33342 (#H342; Dojindo, Kumamoto, Japan), and fluorescence images of wells were acquired using ImageXpress Pico (Molecular Devices). Hoechst 33342-positive cells were counted.

### Animals

All animal procedures were performed in accordance with the protocol approved by the Animal Experimentation Committee of Osaka University. Athymic nude mice (7-week-old male BALB/c Slc-nu/nu mice) were obtained from the Shimizu Laboratory Supplies Co. (Kyoto, Japan) and acclimatised for 1 week prior to experimentation. All mice used in the current study were housed in AAALAC-accredited facilities, and the experimental protocol was approved by the Osaka University Institutional Care and Use Committee (#30-4-6). Experimental rooms were maintained at 21 ± 1°C with 50 ± 20% relative humidity, ventilated with a minimum of 15 HEPA-filtered air changes per hour. Animals were maintained under a 12:12 h light/dark cycle and provided access to food and water *ad libitum*.

### *In vivo* tumour formation analysis

N417 cells were treated with Accutase™ (Innovative Cell Technologies, CA, USA) for 5 min. The cells were collected by centrifugation at 500 *× g* for 3 min. An aliquot of cells, disaggregated by gentle trituration, was used to assess cell viability and number. The cells were suspended in 50 μl of serum-free RPMI-1640 medium, followed by the addition of 50 μl of Matrigel (#354234; Corning, NY, USA). To generate tumour xenografts in mice, N417 cells (5.0 × 10^5^) were implanted subcutaneously into the mid-dorsal region of 7-week-old male nude mice (BALB/c Slc-nu/nu) under isoflurane anaesthesia (#099-06571; FUJIFILM Wako Pure Chemical Co., Osaka, Japan). Tumours were allowed to grow for 2–4 weeks until a tumour size of 150 mm^3^ was achieved. Subsequently, 1.5 μg of L687 in DMSO (0.3 μl) or DMSO (0.3 μl) in 10 μl PBS was administered intratumorally. After 24 h, ASO (10 μg) and 1.5 μg L687 in DMSO (0.3 μl) or DMSO (0.3 μl) were administered intratumorally. Tumours were collected after 24 h under isoflurane anaesthesia. The tumour was immediately immersed in 350 μl RLT Lysis Reagent (#79216; Qiagen, MD, USA) containing 1% 2-mercaptoethanol (FUJIFILM Wako Pure Chemical Co.), and total RNA was extracted as described above. For *in vivo* tumour analysis, long and short diameters were measured, and the tumour volume was calculated as follows : (0.5 × [tumour long diameter] × [tumour minor diameter]^2^).

### Enzyme-linked oligosorbent assay (ELOSA)

Following treatment with ASO and L687, tumours were collected and stored at –80°C. Tissue lysates were obtained by homogenising 50 mg of tissue in RIPA buffer (2.5 μl/mg). The tissue suspension was then diluted with PBS. Streptavidin-coated 96-well plates (#15503; Thermo Fisher Scientific, MA, USA) were washed twice with 120 μl of PBS containing Tween20 (PBS-T; #170-0531; Bio-Rad, CA, USA). A template solution (100 μl) containing SRRM4_ASO _template diluted with PBS-T (1000×) was added to each well, followed by incubation at 37°C for 2 h. A standard curve, using known concentrations of SRRM4_ASO added to the normal tissue lysate, was prepared using a four-parameter logistic analysis method. After washing twice with 120 μl of PBS-T, 100 μl of freshly prepared T4 DNA ligase (#B0202S; New England Biolabs, MA, USA) was added and incubated at 15°C overnight. The wells were then washed twice with SuperBlock blocking buffer (#37580; Thermo Fisher Scientific, MA, USA) prepared in PBS (b-PBS), followed by treatment with 40 μl of S1 nuclease solution (#2410A; Takara Bio, Shiga, Japan) for 30 min. Next, 100 μl of anti-digoxigenin-AP Fab fragment solution (#11093274910; Roche, Buchs, Switzerland) was added and incubated at 37°C for 1.5 h in a humidified incubator under 5% CO_2_. After washing twice with b-PBS, 100 μl of AttophosAP (#S1001; Promega, WI, USA) was added, and the fluorescence emission intensity was measured after 30 min using an Infinite M1000 (Tecan, Männedorf, Switzerland) or a Nivo Multimode Plate Reader (PerkinElmer, MA, USA). Tissue levels of SRRM4_ASO were quantified based on the luminescence intensity using a standard curve.

### Statistical analysis

Each time point was assayed at least in triplicate, and all experiments were performed multiple times to confirm reproducibility. Data are presented as mean ± standard error of the mean (SEM) from three to six independent experiments. Statistical differences were analysed using one-way analysis of variance (ANOVA), followed by Tukey's t-test. The following statistically significant differences were considered: *P < 0.05, **P < 0.01, ***P < 0.001 and ****P < 0.0001.

## Results

### L687 induces Ca^2+^ influx via TRPC3/C6/C7 channels

**Figure 1.**
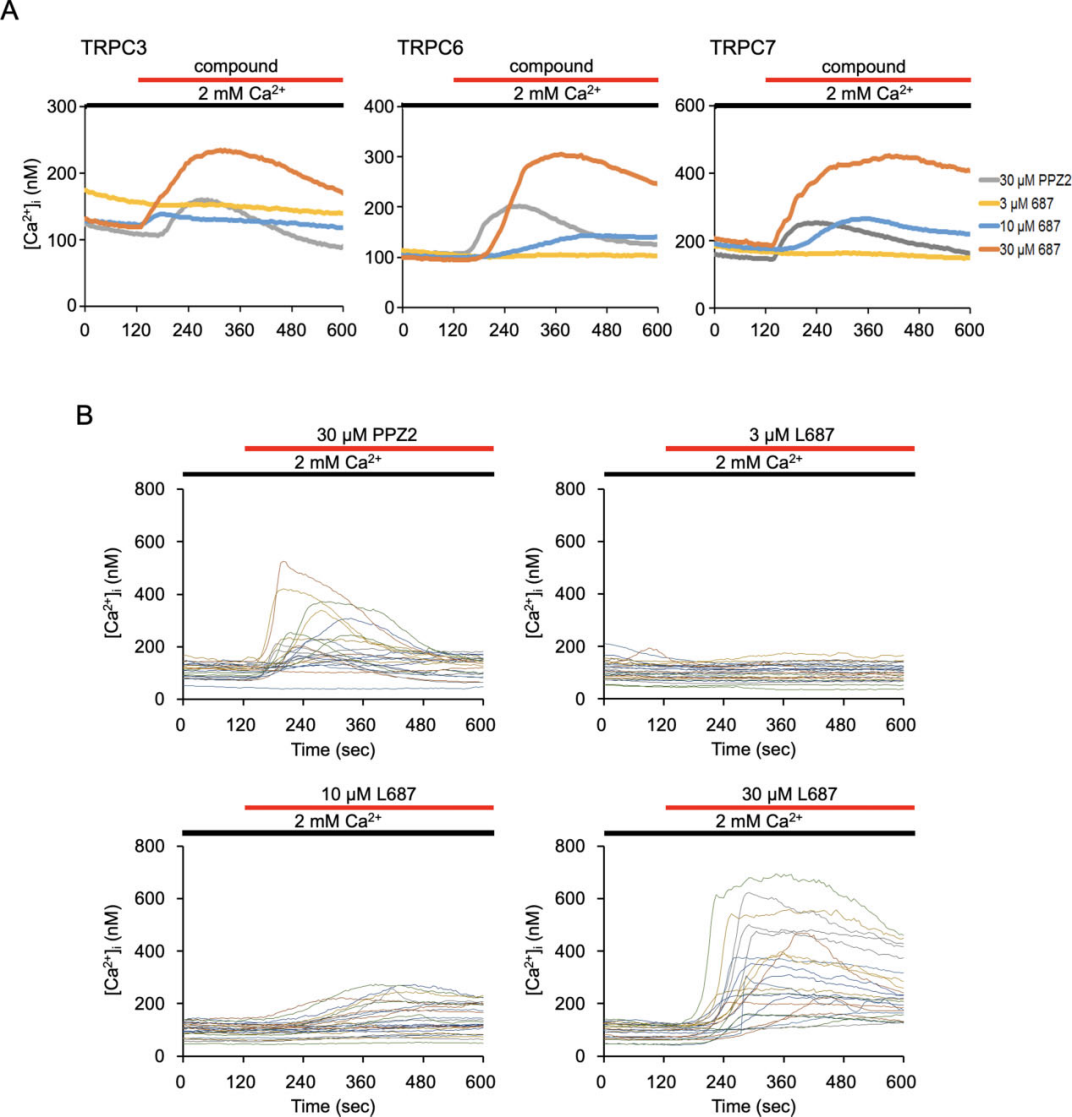
L687, a TRPC3/C6/C7 activator, induces intracellular Ca^2+^ uptake. (**A**) Analysis of intracellular Ca^2+^ influx with various concentrations of L687 or PPZ2 in HEK293 cells, overexpressing TRPC3/C6/C7, respectively, in the presence of 2 mM Ca^2+^. Ca^2+^ influx was recorded following the addition of each compound. (**B**) Analysis of Ca^2+^ influx into the A549 cells in the presence of 2 mM Ca^2+^. After adding PPZ2 (30 μM) or L687 (3, 10 and 30 μM), the Ca^2+^ influx was analysed. TRPC, transient receptor potential canonical.

Calcium imaging was performed to evaluate the ability of L687 to activate TRPCs, which was identified by modifying the PPZ2 structure, using respective TRPC3/C6/C7-expressing cells. PPZ2 is a known selective activator of TRPC3/C6/C7 (28,30). Herein, we observed that L687 dose-dependently increased Ca^2+^ influx via TRPC3/C6/C7 (Figure 1A). Additionally, we examined the selectivity for each TRP channel (TRPC1, C2, C4, C5, A1, V1 and M2) via overexpression in HEK297 cells, which were not activated (Supplementary Figure S2). In A549 cells (TRPC non-overexpressed), treatment with L687 induced Ca^2+^ influx. Notably, the intrinsic activity of 30 μM L687 was higher than that of PPZ2 (Figure 1B).

### L687, a TRPC3/C6/C7 activator, induces cellular uptake of oligonucleotides

**Figure 2.**
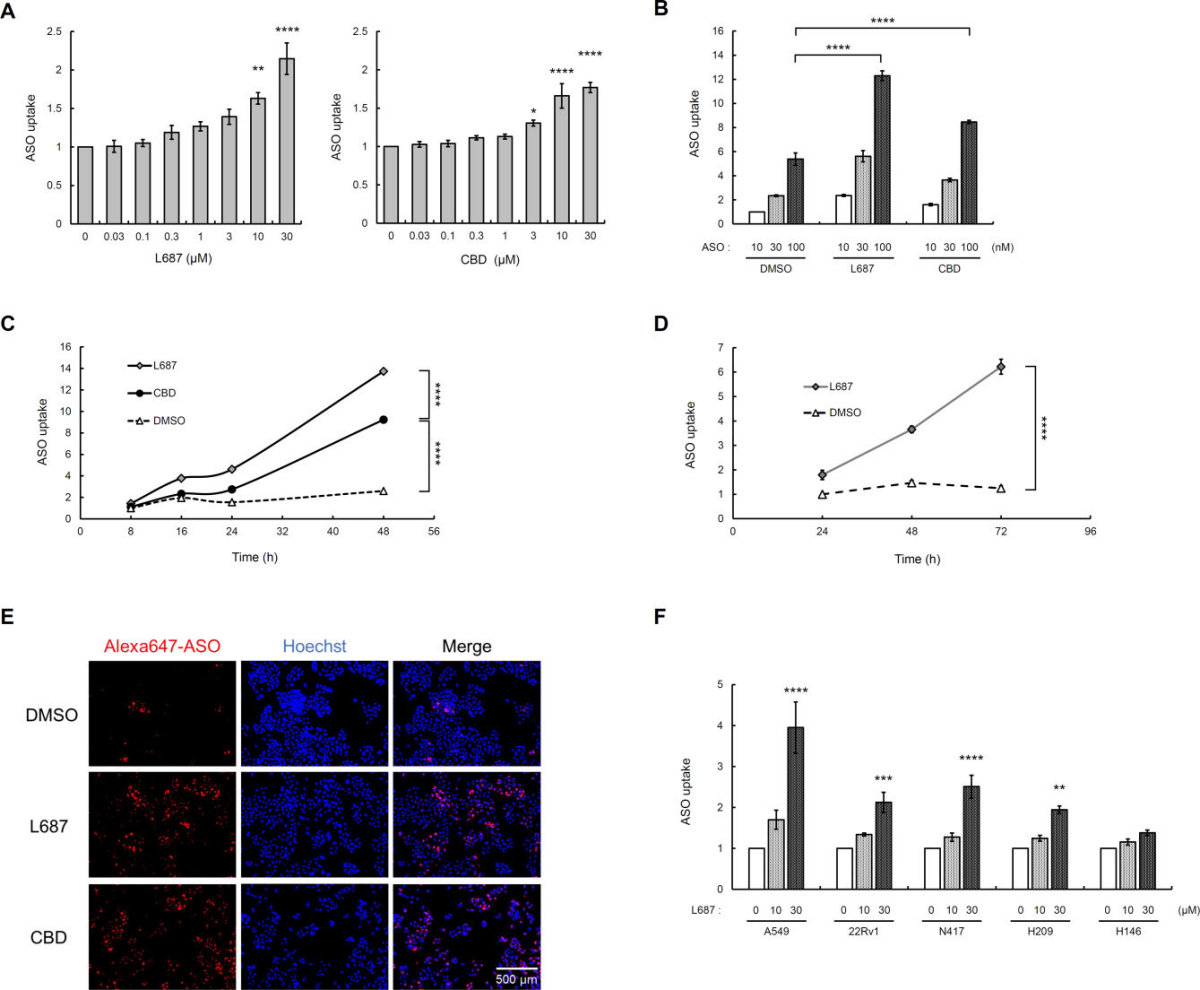
L687 induces intracellular uptake of oligonucleotides. (**A**) Analysis of ASO uptake mediated by L687 and CBD in A549 cells. Alexa647-AmNA#26 (10 nM) with L687 or CBD (0.03–30 μM) was added to the medium, and the intracellular fluorescence intensity was analysed by flow cytometry after 24 h. The relative MFI is shown, compared with that of the medium containing DMSO. Data are presented as mean ± standard error of the mean (SEM) of six independent experiments (*n* = 6). Statistical significance was determined by comparing with values of DMSO using Tukey's test. *P < 0.05, **P < 0.01, ***P < 0.001, ^****^P < 0.0001. (**B**) Analysis of ASO uptake at various concentrations of ASO in the presence of L687 or CBD. Various amounts (10, 30 and 100 nM) of Alexa647-AmNA#26 and L687 or CBD (30 μM) were added to the medium, and the intracellular fluorescence intensity was analysed after 24 h. The relative MFI is presented as the mean ± SEM of three independent experiments (*n* = 3). (**C**) Time course of relative amounts of intracellular ASO (8–48 h). Alexa647-AmNA#26 (10 nM) and L687 or CBD (30 μM) were added to the medium, and intracellular fluorescence intensity was analysed at 8, 16, 24 and 48 h. DMSO was added instead of the activator, and the values are shown relative to the MFI measured at 8 h. Data are presented as mean ± SEM of three independent experiments (*n* = 3). (**D**) Time course of relative amounts of intracellular ASO (24–72 h). Alexa647-AmNA#26 (10 nM) and L687 or CBD (30 μM) were added to the medium, and the intracellular fluorescence intensity was analysed after 24, 48 and 72 h. DMSO was added instead of the activator, and values are shown relative to the MFI measured at 24 h. Data are presented as mean ± SEM of three independent experiments (*n* = 3). (**E**) Fluorescence imaging analysis of ASO incorporated into cells. Alexa647-AmNA#26 (100 nM) and L687 or CBD (30 μM) were added to the medium, and Hoechst staining, fluorescence microscopy imaging, and image analysis were performed after another 48 h. (**F**) Analysis of ASO uptake in various cell lines, including the NSCLC cell line A549, prostate cancer cell line 22Rv1, and SCLC cell lines (N417, H146 and H209). Alexa647-AmNA#26 (10 nM) and L687 (10 and 30 μM) were added to the medium, and intracellular fluorescence intensity was analysed after another 48 h. Values are shown relative to the MFI measure under the addition of DMSO, rather than the activator, in each cell type. Data are presented as mean ± SEM of three independent experiments (*n* = 3). ASO, antisense oligonucleotide; CBD, cannabidiol; DMSO, dimethyl sulfoxide; MFI, mean fluorescence intensity; NSCLC, non-small cell lung cancer; SCLC, small cell lung cancer.

TRPC3/C6, but not TRPC7, were expressed in A549 cells (31), as confirmed by RT-qPCR and western blot (Supplementary Figure S3 and S8). Flow cytometry was performed to measure the cellular uptake of ASO by L687, a mixture of Alexa647-labelled ASO and L687, added to the medium of A549 lung cancer cells, and fluorescence intensity in cells. The ASO used in the current study was previously reported as a negative control that did not impact SRRM4 mRNA (32). Cannabidiol (CBD), a non-selective TRP channel activator, was used as a reference compound for TRP channel activation (33). CBD is also capable of activating TRPC6 channels in a nonspecific manner, as shown in Supplementary Figure S4, although CBD is a known activator of TRPVs and TRPA1. The addition of ASO with L687 to the medium could dose-dependently increase the fluorescence intensity after 24 h (Figure 2A). Various amounts of ASO (10, 30 and 100 nM) and 30 μM L687 were added to the A549 cell medium, increasing ASO uptake significantly (*P* < 0.0001) at 24 h; CBD increased ASO uptake, albeit with lower activation (Figure 2B). Notably, ASO uptake in the presence of L687 or CBD was significantly increased in a time-dependent manner until 48 h (*P* < 0.0001) (Figure 2C), with L687-induced effects on ASO uptake observed until 72 h (Figure 2D). ASOs with L687 were added to the medium of A549 cells, with Hoechst staining performed after 48 h. Subsequently, images of Alexa647-ASO were analysed by fluorescence microscopy, with red indicating ASO and blue indicating cell nuclei, suggesting that the amount of intracellular ASO increased in the presence of L687 or CBD, similar to the flow cytometry results (Figure 2E). The L687-mediated intracellular ASO uptake was analysed in non-small cell lung cancer cells A549, prostate cancer cells 22Rv1, and small cell lung cancer (SCLC) cells (N417, H146 and H209) using flow cytometry after 48 h. The addition of L687 significantly increased the amount of intracellular ASO in all examined cell lines except for H146, which showed a slightly lower level of intracellular ASO (Figure 2F).

### Evaluation of ASO activity following incubation with L687

**Figure 3.**
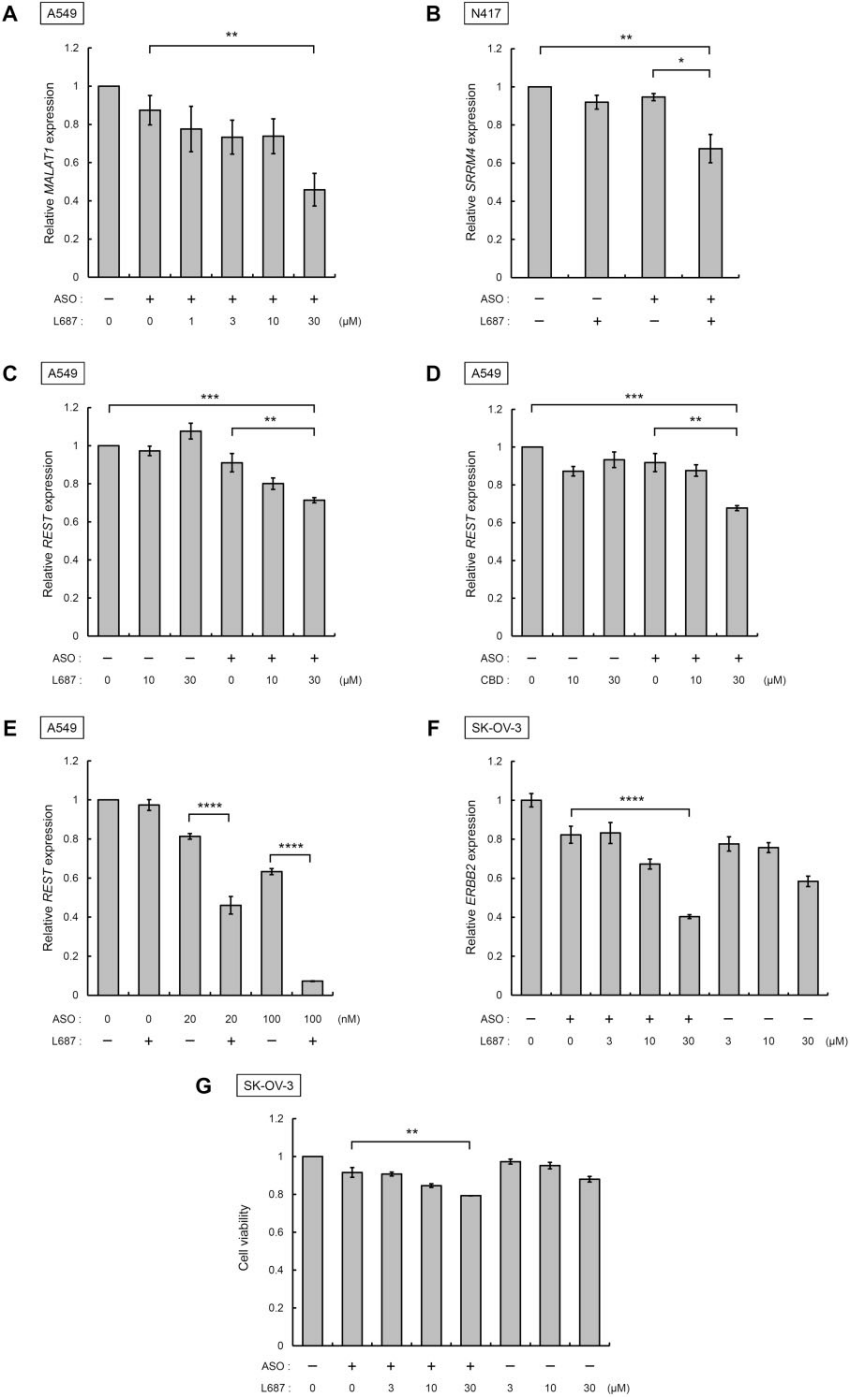
Effects of the TRPC3/C6/C7 activator L687 or CBD on ASO activity. (**A**) Analysis of *MALAT1* expression by adding MALAT1_ASO with L687. MALAT1_ASO (100 nM) was added to either 1–30 μM L687 in the medium. A549 cells were collected after 48 h, and total RNA was prepared for RT-qPCR analysis. The relative expression of *REST* versus *GAPDH* was compared with that of *MALAT1* in untreated A549 cells. All data are presented as mean ± standard error of the mean (SEM) of three independent experiments (*n* = 3). Statistical significance was determined by comparing with values of mock DMSO using Tukey's test. *P < 0.05, **P < 0.01, ***P < 0.001, ^****^P < 0.0001. (**B**) Analysis of *SRRM4* expression by adding SRRM4_ASO with L687. SRRM4_ASO (100 nM) was added with 30 μM L687 to the medium. N417 cells were collected after 48 h, and total RNA was prepared for RT-qPCR analysis. The relative expression of *SRRM4* versus *GAPDH* was compared with that of *SRRM4* in untreated N417 cells. (**C**, **D**) Analysis of *REST* expression by adding REST_ASO with L687. REST_ASO (100 nM) was added to 10 and 30 μM of L687 or CBD in the medium. A549 cells were collected after 48 h, and the relative expression of *REST* versus *GAPDH* was analysed. (**E**) Analysis of *REST* expression by adding REST_ASO with L687. REST_ASO (20 and 100 nM) was added to 30 μM L687 in the medium. A549 cells were collected after 72 h, and the relative expression of *REST* versus *GAPDH* was analysed. (**F**) Analysis of *ERBB2* expression by adding ERBB2_ASO with L687. ERBB2_ASO (100 nM) was added to 3–30 μM of L687 in the medium. SK-OV-3 cells were collected after 48 h, and the relative expression of *ERBB2* versus *β-actin* was analysed. (**G**) Viability analysis of SK-OV-3 by adding ERBB2_ASO with L687. ERBB2_ASO (100 nM) was added to 3–30 μM of L687 in the medium. Cell viability was analysed after 48 h of incubation. ASO, antisense oligonucleotide; CBD, cannabidiol; DMSO, dimethyl sulfoxide; RT-qPCR, reverse transcription-quantitative PCR; TRPC, transient receptor potential canonical.

To evaluate the gene knockdown activity of ASOs incorporated into cells by L687, we analysed expression levels of MALAT1 in A549 cells using RT-qPCR. We used a MALAT1-targeted ASO (MALAT1_ASO). Compared with MALAT1_ASO without L687, the addition of MALAT1_ASO with 30 μM L687 significantly decreased MALAT1 expression in A549 cells (*P* < 0.01; Figure 3A). We have previously developed an SRRM4-targeted ASO (SRRM4_ASO) that reduces SRRM4 expression in SCLC and neuroendocrine prostate cancer cells (32,34). We evaluated the effect of L687 on SRRM4_ASO activity in N417 cells. After 48 h of culture, SRRM4_ASO with L687 significantly decreased the relative expression of SRRM4 when compared with treatment with SRRM4_ASO alone (Figure 3B). Furthermore, we determined the expression of REST in A549 cells. A549 cells were selected to express high levels of REST (35). For functional analysis of ASO, we employed the REST-targeted ASO (REST_ASO) developed in our laboratory. REST_ASO (100 nM), with or without L687 or CBD, was added to the medium, and A549 cells were collected after 48 h of culture. Total RNA was prepared and used for RT-qPCR. The addition of ASO with L687 (Figure 3C) or CBD (Figure 3D) significantly decreased the relative expression of REST when compared with that in untreated A549 cells or cells treated with ASO alone. L687 and CBD without REST_ASO did not affect REST expression. We further extended the incubation time after adding REST_ASO and L687 to A549 cells. The relative expression of REST mRNA after 72 h was significantly repressed when compared with that observed after 48 h (Figure 3E). Thus, the TRPC3/C6/C7 activator L687 induced the intracellular uptake of REST_ASO into A549 cells, followed by the repression of REST expression. Similar results were obtained using other ASOs targeting ERBB2 (ERBB2_ASO) (Figure 3F). The addition of ERBB2_ASO with 30 μM L687 significantly reduced the relative expression of ERBB2 when compared with ERBB2_ASO without L687 (*P* < 0.0001). ERBB2_ASO with L687 was added to SK-OV-3 cells, and cell viability was analysed after 48 h. The addition of ERBB2_ASO with 30 μM L687 significantly reduced (*P* < 0.01) cell viability when compared with ERBB2_ASO without L687.

### Effect of TRPC3/C6 expression and Ca^2+^ influx on L687-mediated ASO uptake

**Figure 4.**
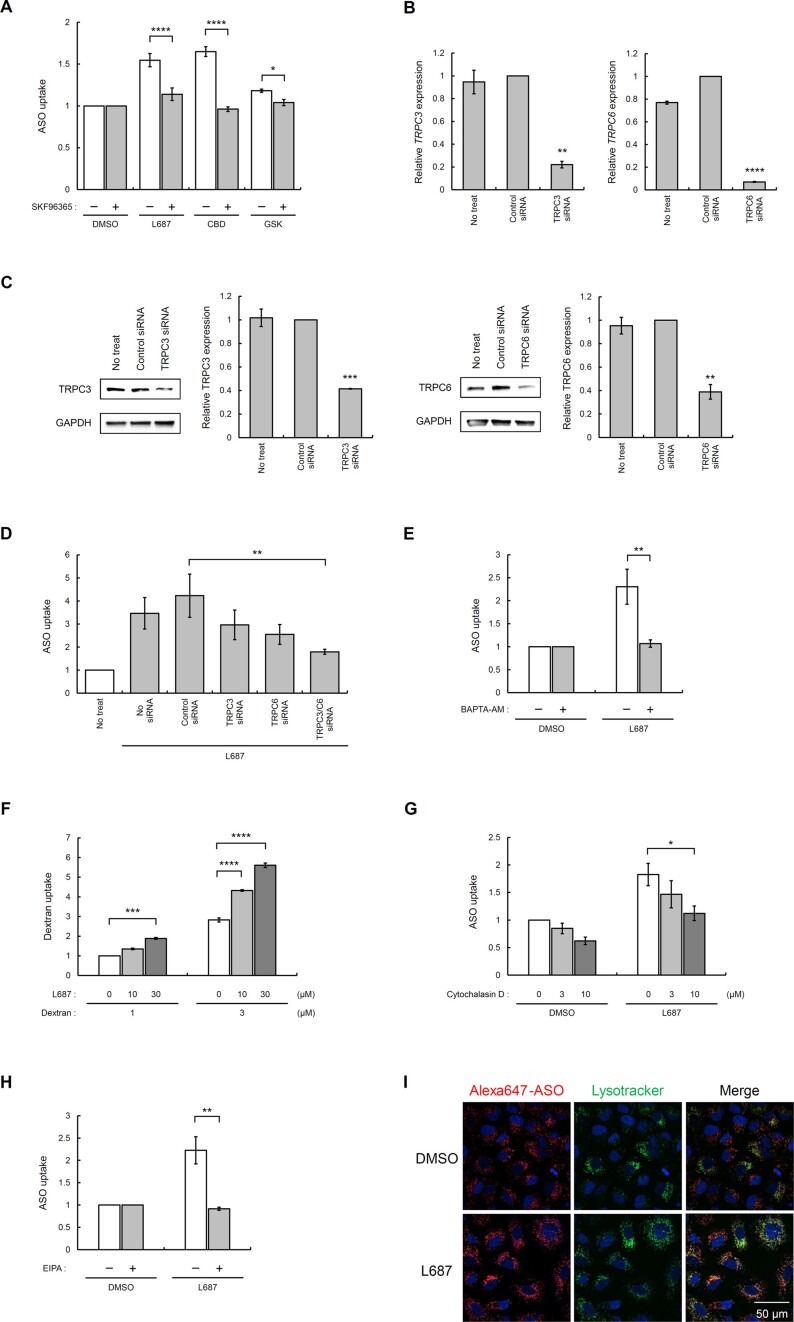
Effects of the TRPC3/C6/C7 inhibitor, knockdown of TRPC3/C6, and Ca^2+^ chelator on ASO uptake, and examination of L687-mediated uptake pathways. (**A**) ASO uptake was analysed by incubating cells with or without a TRPC inhibitor (SKF96365). Alexa647-AmNA#26 (10 nM) was added to either 10 μM L687, 20 μM GSK1702934A, or 30 μM CBD with or without 20 μM SKF96365 in the medium. After 24 h, the intracellular fluorescence intensities were analysed by flow cytometry. Data are shown as the relative MFI of ASO in DMSO. All data are presented as mean ± standard error of the mean (SEM) of three independent experiments (*n* = 3). Statistical significance was determined by comparing with values of DMSO using Tukey's test. *P < 0.05, **P < 0.01, ***P < 0.001, ^****^P < 0.0001. (**B**) siRNA-mediated knockdown of the TRPC3/C6 channels. TRPC3 siRNA (30 nM) or TRPC6 siRNA (10 nM) was transfected into A549 cells using Lipofectamine3000, and cells were collected after 48 h. The relative expression of *TRPC3/C6* was analysed and compared with that in the untreated cells. (**C**) siRNA-mediated knockdown of TRPC3/C6 channel. TRPC3 siRNA (30 nM) or TRPC6 siRNA (10 nM) was transfected into A549 cells using Lipofectamine3000, and cells were collected after 48 h. The relative expression of TRPC3/C6 was analysed by western blot and compared with that in untreated cells. (**D**) The effects of siRNA-mediated TRPC3/C6 channel knockdown on ASO uptake. TRPC3 siRNA (30 nM), TRPC6 (10 nM), or a combination of both were transfected into A549 cells for 48 h. The medium was replaced with Alexa647-AmNA#26 containing L687, and intracellular fluorescence intensities were analysed after 24 h. Data are shown as the relative MFI of ASO in DMSO. (**E**) Analysis of ASO uptake after incubating cells with a Ca^2+^ chelator (BAPTA-AM). ASO and L687 were added to the medium, with or without 10 μM BAPTA-AM. After 24 h, intracellular fluorescence intensities were analysed by flow cytometry. (**F**) Analysis of dextran uptake mediated by L687. Alexa647-labelled dextran (1 and 3 μM) with 10 and 30 μM L687 was added to the medium, and A549 cells were cultured for 24 h. Intracellular fluorescence was analysed by flow cytometry, and the relative MFI was compared with 1 μM of Alexa647-dextran with DMSO. (**G**) Analysis of ASO uptake by incubating the cells with a macropinocytosis inhibitor (Cytochalasin D). L687 (30 μM) was then added to the medium. The following day, cells were then washed twice with PBS and incubated with cytochalasin D in the medium for 1 h. Then, cells were washed twice with PBS and incubated with Alexa647-AmNA#26 (10 nM) and L687 (30 μM). After 4 h, intracellular fluorescence intensities were analysed by flow cytometry. (**H**) Analysis of ASO uptake by incubating cells with a macropinocytosis inhibitor (EIPA). ASO and L687 were added to the medium with or without 100 μM EIPA. After 24 h, intracellular fluorescence intensities were analysed by flow cytometry. (**I**) Fluorescence imaging analysis of ASO incorporated into cells. Alexa647-AmNA#26 (100 nM) and L687 (30 μM) were added to the medium, and staining with Lysotracker-green and Hoechst, fluorescence microscopy imaging, and image analysis were performed after 48 h. ASO, antisense oligonucleotide; CBD, cannabidiol; DMSO, dimethyl sulfoxide; MFI, mean fluorescence intensity; TRPC, transient receptor potential canonical.

To analyse the relationship between TRPC3/C6 expression and ASO uptake mediated by L687, we used a TRPC inhibitor (SKF96365) to inhibit Ca^2+^ influx via TRPC (36,37). GSK1702934A, another TRPC3/C6 channel activator, has also been used (38). Various TRPC3/C6 activators and ASO were added to the medium containing DMSO or SKF96365. After 24 h of culture, the intracellular fluorescence intensity was measured by flow cytometry. The addition of L687 or CBD increased the relative level of intracellular ASO to a greater extent than GSK1702934A. The addition of SKF96365 suppressed the cellular uptake of ASO (Figure 4A). We analysed ASO uptake by repressing TRPC3 or TRPC6 expression using respective siRNAs. Forty-eight hours after siRNA transfection, TRPC3 and TRPC6 expressions were analysed using RT-qPCR (Figure 4B) and western blot analysis (Figure 4C). Both mRNA and protein analyses revealed significant and specific siRNA-mediated knockdown of TRPC3 and TRPC6. Next, A549 cells were transfected with each siRNA for 48 h, and the medium was replaced with a medium containing ASO with L687. After another 24 h of incubation, ASO uptake mediated by L687 and negative control siRNA was increased, similar to that observed in untreated cells, whereas siRNAs targeting TRPC3 and TRPC6 exhibited decreased L687-mediated intracellular ASO uptake (Figure 4D). These results suggest that Ca^2+^ influx via TRPC3/C6 channels is essential to enhance the cellular uptake of ASOs. To analyse the role of Ca^2+^ in the uptake-enhancing effect of the activator, a Ca^2+^ chelator (BAPTA-AM) was used (Figure 4E) (39). The addition of BAPTA-AM significantly suppressed L687-mediated ASO uptake (*P* < 0.01). To determine the contribution of L-type voltage dependent Ca^2+^ channels to ASO uptake (56), we co-administered the selective L-type Ca^2+^ channel blocker nifedipine with L687 to A549 cells in the presence of ASOs. Under these conditions, nifedipine did not affect L687 mediated ASO uptake (Supplementary Figure S7). To address the mechanism underlying the cellular uptake of ASO by TRPC activators, we expanded the use of Alexa647-labelled dextran, an endosomal marker, to evaluate cellular uptake (40). Alexa647-labelled dextran with L687 was added to the medium of A549 cells, and the fluorescence intensity was measured flow cytometrically after 24 h. L687 promoted the uptake of Alexa647-labelled dextran in a dose-dependent manner (0, 10, and 30 μM) (Figure 4F). Accordingly, these results suggest that L687 causes Ca^2+^ influx via TRPC3/C6 channels and promotes the uptake of polysaccharides other than ASO. Next, we examined the effect of EIPA, which inhibits macropinocytosis, on L687-mediated effects (Figure 4H). EIPA inhibited L687-mediated uptake of ASOs. Furthermore, we tested cytochalasin D, which depolymerises actin filaments to inhibit macropinocytosis and phagocytosis, and confirmed that this also inhibited the L687 dependent ASO uptake (Figure 4G). These results implied that L687 might induce macropinocytosis (41–43). In the cytochalasin D experiments, A549 cells were preincubated with L687 for 24 h, followed by treatment with or without cytochalasin D for 1 h after L687 was washed out. After cytochalasin D was washed out, ASOs and L687 were treated for 4 h. Under these conditions, ASO uptake occured within 4 h in the absence of Cytochalasin D. This means that L687 mediated ASO uptake is relatively rapid if TRPC3/C6 channels have sufficiently been activated by L687. Moreover, images of Alexa647-ASO were analysed by fluorescence microscopy, where ASOs are indicated in red, lysosomes in green, and cell nuclei in blue (Figure 4I). Supplementary Figure S6 shows the z-axis scanning images of A549 cells treated with Alexa647-ASO in the presence or absence of L687. These images demonstrated that abundant amounts of ASOs in the presence of L687 were incorporated into cells compared to those in the absence of L687 and that a major fraction of ASOs was located in endosomes/lysosomes and a part of ASOs migrated into the nucleus with L687.

### Effects of Ca^2+^ in the medium and UNC7938 on ASO trafficking mediated by L687

**Figure 5.**
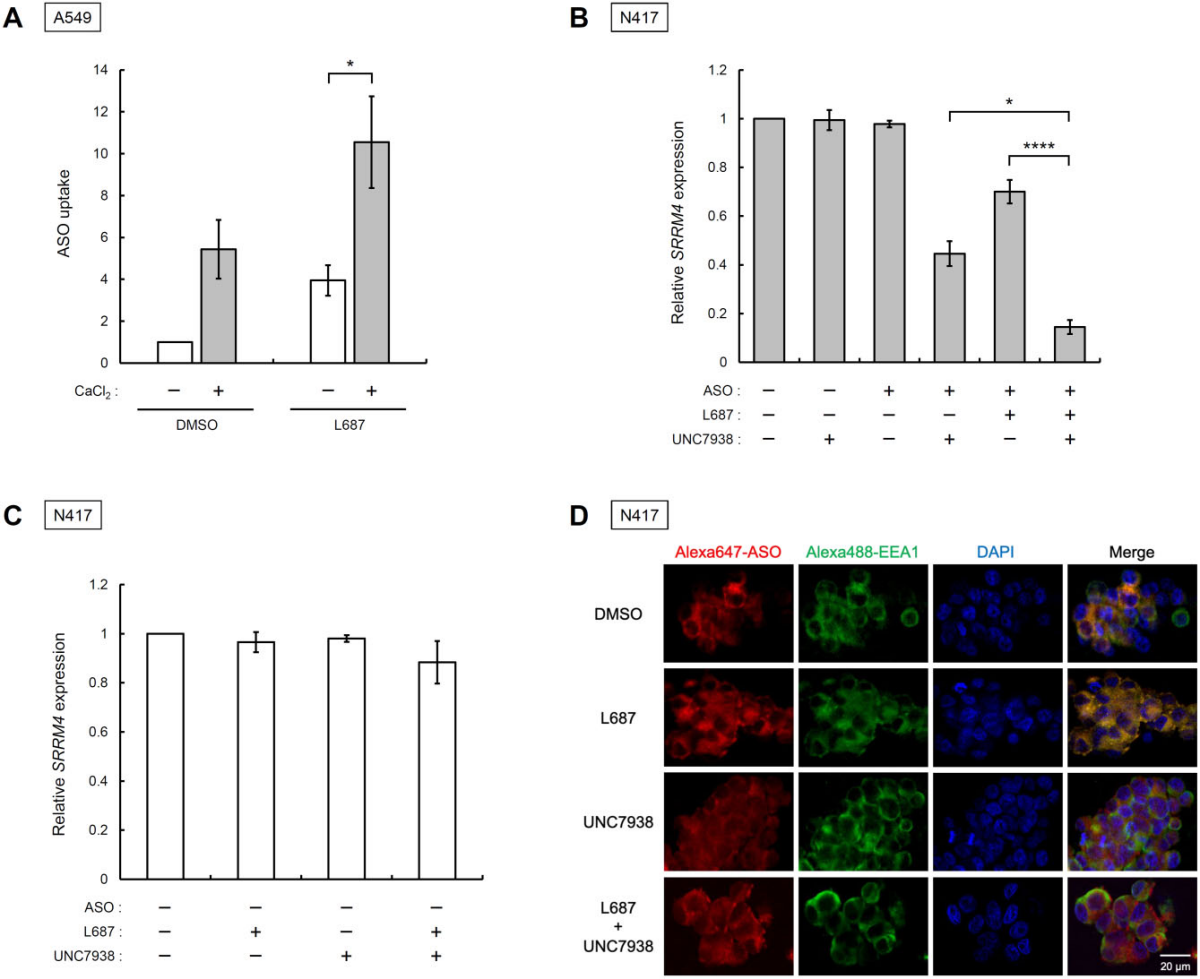
Effects of L687 in the presence of Ca^2+^ in the medium and UNC7938, an endosomal escape enhancer. (**A**) Analysis of ASO uptake using the CEM method and L687. Alexa647-AmNA#26 (10 nM) and 30 μM L687 were added to medium containing 0 or 9 mM CaCl_2_. Intracellular fluorescence intensities were analysed using flow cytometry after 48 h of A549 cell culture. The data are shown as the relative MFI of ASO in DMSO. All data are presented as mean ± standard error of the mean (SEM) of three independent experiments (*n* = 3). Statistical significance was determined by comparing with values of mock DMSO using Tukey's test. *P < 0.05, **P < 0.01, ***P < 0.001, ^****^P < 0.0001. (**B** and **C**) Analysis of *SRRM4* expression after addition of SRRM4_ASO, L687, and UNC7938. N417 cells were cultured in a medium containing 100 nM SRRM4ASO and 30 μM L687. After 48 h of culture, the medium was replaced with medium containing 2 μM UNC7938. After culturing for 24 h, cells were collected, and total RNA was prepared for RT-qPCR. The relative expression of *SRRM4* versus *GAPDH* was compared with that of *SRRM4* in untreated N417 cells. (**D**) Fluorescence imaging analysis of ASO incorporated into cells. N417 cells were cultured in a medium containing 100 nM Alexa647-AmNA#26 and 30 μM L687. After 48 h of culture, the medium was replaced with medium containing 2 μM UNC7938. After culturing for 24 h, immunofluorescence staining, fluorescence microscopy, and image analysis were performed. ASO, antisense oligonucleotide; DMSO, dimethyl sulfoxide; MFI, mean fluorescence intensity; RT-qPCR, reverse transcription-quantitative PCR.

L687 promoted ASO uptake, possibly by inducing endocytosis following Ca^2+^ influx via TRPC3/C6 channels. We have previously developed a CEM method for the efficient intracellular uptake of oligonucleotides using high Ca^2+^ concentrations in the culture medium. We further investigated the effect of L687 on ASO uptake under elevated Ca^2+^ concentrations. A CaCl_2_ solution was added to the medium to achieve a final concentration of 9 mM, followed by the addition of ASO with L687. Fluorescence intensity was measured after 48 h of culture using flow cytometry. L687 increased ASO uptake even at the optimal concentration of the CEM method (Figure 5A). These results suggest that the CEM and L687 methods may promote uptake via Ca^2+^ influx. Given that UNC7938 has been shown to improve oligonucleotide activity by destabilising the endosomal membrane and promoting endosomal escape without participating in cellular uptake (44–46), we examined whether combining UNC7938 and L687 could further elicit ASO activity. ASO with L687 was added to the medium and cultured for 48 h; the medium was then replaced with a medium containing UNC7938. N417 cells were collected after 24 h, and SRRM4 expression was analysed by RT-qPCR. The addition of UNC7938 in combination with ASO and L687 reduced SRRM4 expression (Figure 5B), suggesting that the endosomal escape of ASO by UNC7938 was improved, thereby enhancing knockdown activity. Alexa647-labelled ASO was used to analyse the effect of UNC7938 on intracellular ASO trafficking. Neither L687 nor UNC7938 affected the relative expression of SRRM4 (Figure 5C). N417 cells were labelled with the endosomal marker EEA-1, and cells treated with Alexa647-labelled ASO were imaged using fluorescence microscopy (Figure 5D), with ASO indicated in red, endosomes in green, and nuclei in blue; yellow fluorescence indicated ASO accumulation predominantly in endosomes without UNC7938 (Figure 5D). In the presence of UNC7938, ASO migrated into the nucleus and cytosol, suggesting that UNC7938 increased endosomal escape of ASO, thereby enhancing knockdown activity.

### L687-mediated ASO uptake in tumours in a xenograft mouse model

**Figure 6.**
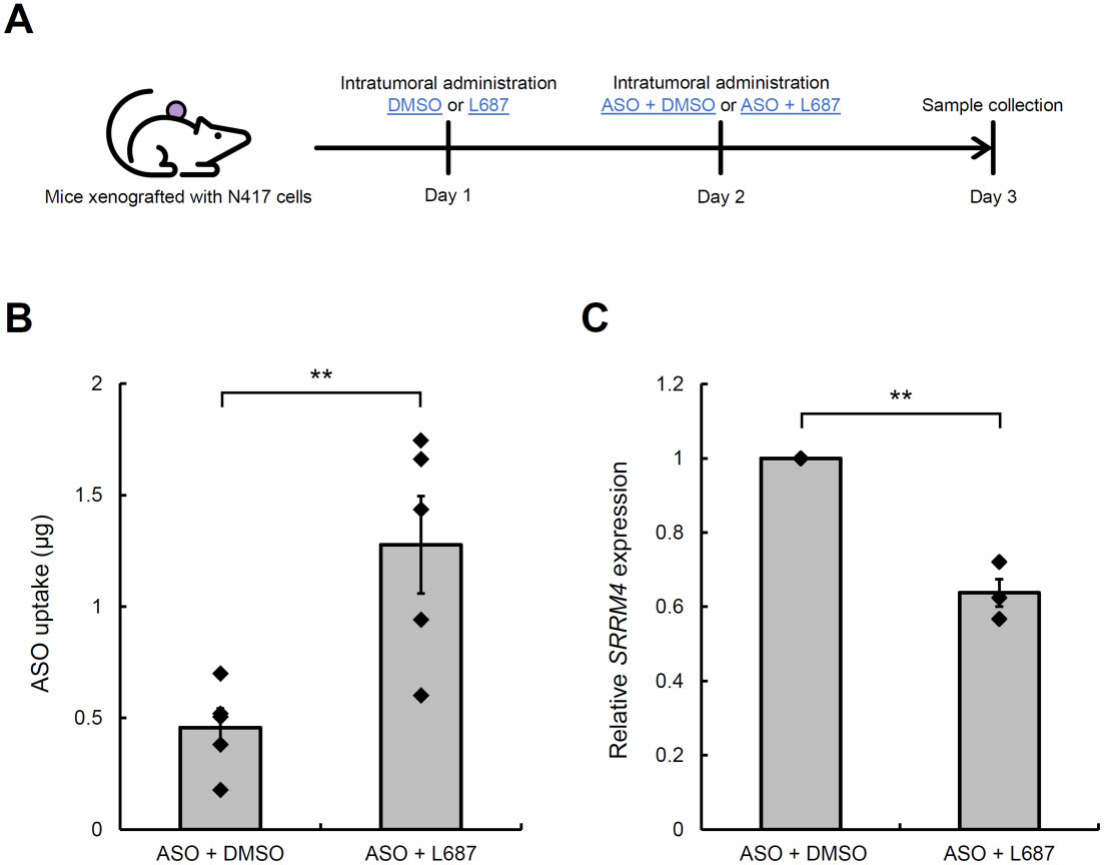
Effects of L687 on ASO uptake and activity in tumours of xenograft mice. N417 cells (5.0 × 10^5^) were subcutaneously implanted into 8-week-old BALB/c Slc-nu/nu mice (*n* = 5). Fourteen days later, DMSO or L687 was administered intratumorally at 1.5 μg, followed by SRRM4_ASO (10 μg) with DMSO or L687 (1.5 μg) 24 h later. (**A**) Experimentscheme. (**B**) The amount of SRRM4_ASO analysed using sequence-specific ELOSA. Statistical significance was determined by comparing with values for NT using one-way analysis of variance (ANOVA), followed by Dunnett's *t*-test. **P < 0.01. (**C**) Relative expression of *SRRM4* in tumours was analysed by RT-qPCR. ASO, antisense oligonucleotide; DMSO, dimethyl sulfoxide; ELOSA, enzyme-linked oligosorbent assay; RT-qPCR, reverse transcription-quantitative PCR.

To further analyse ASO uptake *in vivo*, we examined the effects of L687 *in vitro*, using xenograft mice transplanted with human cancer cells. First, N417 cells were transplanted into xenograft mice. SRRM4_ASO (10 μg) and L687 (1.5 μg) were then administered intratumorally, and tumours were harvested after 24 h. The amount of ASO in the tumour was analysed using sequence-specific ELOSA (47). Tumours treated with L687 plus SRRM4_ASO exhibited significantly higher SRRM4_ASO levels than those treated with DMSO plus SRRM4_ASO (Figure 6A). Relative expression of *SRRM4* was significantly reduced in tumours administered SRRM4_ASO plus L687 when compared with expression levels in tumours administered SRRM4_ASO with DMSO (Figure 6B).

## Discussion

Oligonucleotide therapeutics, such as ASOs and siRNAs, can directly regulate protein expression, which cannot be achieved with small-molecule drugs and antibodies, and have been recognised as next-generation therapeutics. However, the selective delivery of oligonucleotides to target cells and their emerging activities remain major concerns, restricting their applicability. In 2013, the FDA approved mipomersen as an antisense drug for systemic administration to treat familial hypercholesterolemia. However, the liver was the target organ. In 2016, an exon-skipping morpholino oligomer, antisense eteprilsen, targeting the skeletal muscle cells was marketed to treat Duchenne muscular dystrophy; however, its effectiveness remains controversial. In 2016, a splice-switching oligonucleotide, nusinersen, was marketed and found to be beneficial in patients with spinal muscular atrophy who could not access effective medications; however, these patients required intrathecal injection. In 2017, the siRNA drug patisiran was approved for hereditary ATTR amyloidosis; however, it is a lipid nanoparticle formulation targeting the liver. In 2019, givosiran, a ligand-conjugated siRNA, was approved to treat acute porphyria but also targeted the liver. Accordingly, all currently available oligonucleotide therapeutics, including those not listed, are revolutionary and beneficial for patients with uncurable diseases but are limited owing to formulations warranting focal injection or targeting the liver, exhibiting insufficient efficacy in cases where organs other than the liver need to be targeted via systemic injection. Hence, we believe that improved oligonucleotide incorporation into target cells could, at least partially, resolve challenges in systemic oligonucleotide delivery to target organs other than the liver.

The substantial increase in the amount of ASO in the presence of L687 (10 and 30 μM) and CBD (10 and 30 μM) could be attributed to the marked Ca^2+^ influx into cells observed at these activator concentrations. We believe that the higher amount of ASO incorporated by L687 than that by CBD was due to the difference in activation capacity. It is speculated that cellular uptake of ASO induced by activator-mediated Ca^2+^ influx would be delayed. These results suggest that ASO and L687 do not necessarily need to be added simultaneously and that uptake may be enhanced depending on the time elapsed after Ca^2+^ influx induced by L687 (Supplementary Figure S5). Differences in L687-mediated effects on different cell types, including prostate cancer and SCLC cells from different tissues of origin, may be due to differences in channel expression levels, cell morphology and characteristics. The lack of substantial effects in H146 cells could be attributed to the low expression of TRPC3 and C6 channels.

Herein, our experiments revealed that treatment with L687 could increase ASO uptake in concentration- and time-dependent manners. Therefore, we measured knockdown activity using ASO and activators and found that L687 or CBD also increased activity in a concentration- and time-dependent manner. This finding was attributed to an increase in the amount of ASO taken up intracellularly, followed by nuclear translocation. A similar trend of L687-mediated increased RNA repression activity was also observed when *MALAT1*-targeted ASOs were applied to A549 cells and *ERBB2*-targeted ASOs were applied to SKOV3 cells; *REST*, *MALAT1* and *ERBB2*-targeted ASOs used LNA (48–50), and *SRRM4*-targeted ASOs used AmNA (51,52) as artificial nucleic acids. Accordingly, these findings suggest that the addition of L687 can improve the intracellular translocation and activity of oligonucleotides regardless of their sequence and modifications.

Suppression of ASO uptake in the presence of the TRPC channel-selective inhibitor SKF96365 suggests that Ca^2+^ influx via TRPC is associated with ASO uptake. Moreover, SKF96365 abolished the ASO uptake increased by CBD, which is known to activate TRPVs and TRPA1 in A549 cells expressing TRPV1 and TRPA1, as well as TRPC3 and TRPC6 (53,54). In addition, ASO uptake was not affected by nifedipine, a selective blocker of L-type voltage dependent calcium channels, that are widely expressed in various cells as a major source of calcium entry (55,56). These results suggest that Ca^2+^ influx, particularly via TRPC, is required to promote ASO uptake. Onohara *et al.* reported that TRPC3/C6 indirectly mediated NFAT-mediated hypertrophic responses through L-type Ca^2+^ channels in cardiac myocytes, as evidenced by inhibition of angiotensin II-induced NFAT activation by both TRPC blocker, SKF96365 and L-type Ca^2+^ channel blocker, nicardipine (57). On the other hand, we observed that TRPC3/C6 mediated endocytosis of ASOs was not affected by L-type Ca^2+^ channel blocker, nifedipine. Our findings underscore the idea that TRPC3/C6 channels are closely coupled to the endocytic machinery. Next, when siRNA was used to knock down TRPC3 and TRPC6, the effect of L687 was reduced. As A549 cells did not express TRPC7, we focused on TRPC3/C6. Based on the observed findings, L687 may be effective as a drug delivery system (DDS) for the selective delivery of ASO to lung or prostate cancer tissues, given that L687 was thought to promote the selective uptake of ASO in cells with high TRPC3/C6 expression. Moreover, the addition of the Ca^2+^-selective chelator BAPTA-AM suppressed the effect of L687, suggesting that Ca^2+^ plays an important role in the L687-mediated uptake of ASOs. Notably, in addition to ASOs, L687 could incorporate dextran, a different modality from that of oligonucleotides, into cells, and the cellular uptake by TRPC3/C6 channel activation might be a general phenomenon. Furthermore, EIPA and cytochalasin D, which are macropinocytosis inhibitors, suppressed the L687-mediated increase in ASO uptake, suggesting that L687 may improve ASO intracellular uptake by promoting macropinocytosis. However, further investigations are warranted to elucidate the mechanism of cellular uptake via TRPC3/C6 activation.

The effect of L687 on mice was confirmed using SRRM4_ASO, which has been evaluated *in vivo*. Given that the toxicity of L687 has not been comprehensively investigated, experiments were first conducted using intratumoral administration. Both the increased amount and activity of ASO in the tumour well correlated with the *in vitro* findings. Owing to the experimental circumstances, we could not undertake qPCR evaluation on tumours without ASO; however, we compared the results using tumours from the saline group collected in a separate experiment. Although L687-induced knockdown needs to be considered, the *in vitro* results showed that L687 did not alter *SRRM4* expression in N417 cells, suggesting L687 itself is not responsible for *SRRM4* knockdown in tumours.

In the current study, we examined the newly synthesised TRPC3/C6/C7 activator, L687. Our findings confirmed that combining ASO and L687 increased cellular uptake and enhanced the efficacy of ASO. Various small-molecule compounds, such as UNC7938, have been shown to enhance ASO activity by improving endosomal escape or by affecting the endosomal pathway (44,58). However, to the best of our knowledge, no small-molecule compounds similar to L687, capable of inducing the translocation of extracellular oligonucleotides into cells, have been reported. We addressed the mechanism of action of ASO uptake mediated by L687 and suggested that Ca^2+^influx induced efficient ASO uptake into cells. Furthermore, we demonstrated that activation of TRPC3/C6 channels with a specific ligand, such as L687, could facilitate the cellular uptake of oligonucleotides. TRPC3/C6 channels are highly expressed in certain tumour cells, including lung cancer cells. Therefore, the administration of L687 or other TRPC3/C6 activators could facilitate ASO delivery specifically into targeted tumour cells. This novel concept would increase the therapeutic applicability of ASOs and accelerate the overall development of ASOs with/without DDS.

## Supplementary Material

gkae245_Supplemental_File

## Data Availability

The data underlying this article are available in the article and online supplementary material.
