# Supplementary material for: A novel transient receptor potential C3/C6 selective activator induces the cellular uptake of antisense oligonucleotides
Source: Nucleic Acids Res. 2024 Apr 16;52(9):4784–98. doi: 10.1093/nar/gkae245 (PMC11109983; doi:10.1093/nar/gkae245)
Supplement: gkae245_Supplemental_File [file gkae245_supplemental_file.docx]

**Supplemental Data**

**Supplementary Figure 1.**

**
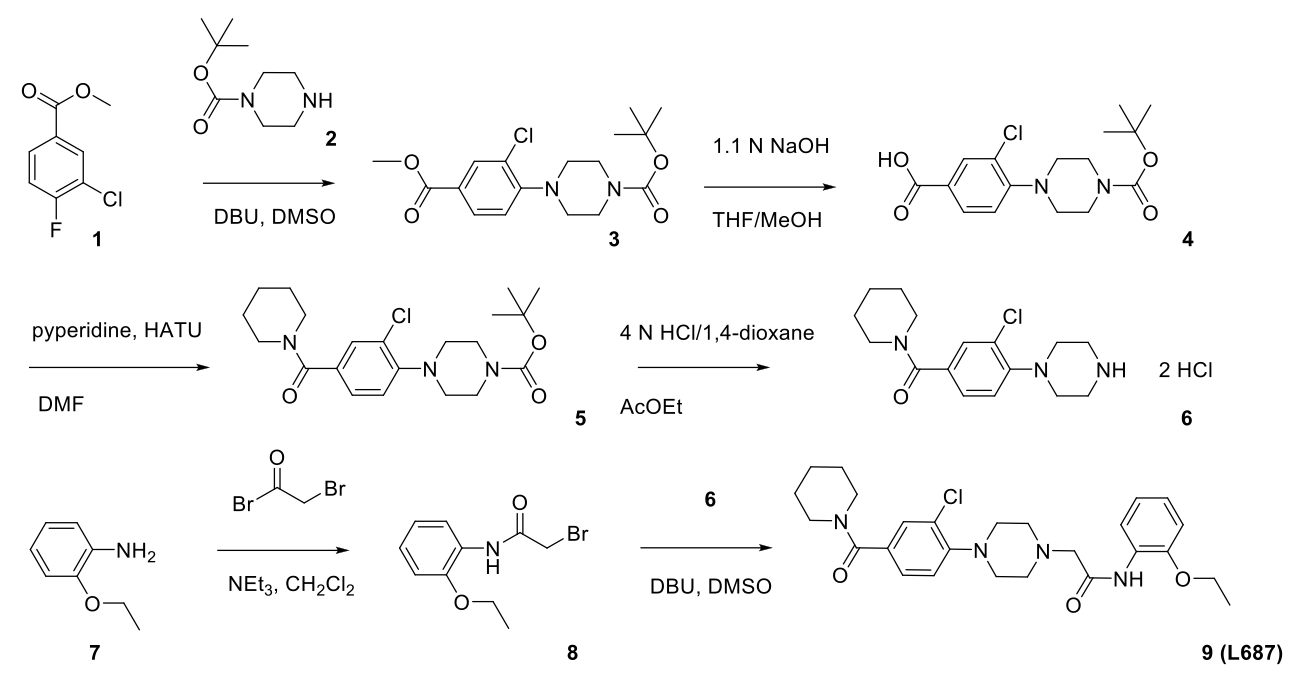
**

General procedures.

­^1^H NMR spectra were recorded on a JEOL JNM-ECZ400S spectrometer and calibrated using a residual undeuterated solvent as an internal reference (CDCl_3_: 7.26 ppm and DMSO-d_6_: 2.50 ppm). LC/ESI-MS data were obtained on a Waters Acquity UPLC H class/QDa system, using a BEH C18 column (1.7 µm, 2.1 × 50 mm), with the following mobile phase: A = 0.1% formic acid, B = acetonitrile, applying a gradient: 0 min/10% B, 3 min/95% B, 3.5 min/ 10% B, 5 min/10% B at a flow rate of 0.5 mL/min, and ionisation was achieved either by positive or negative modes. Thin-layer chromatography was performed on silica gel glass-backed plates (5719, Merck & Co.). Flash column chromatography was conducted using Smart Flash, EPCLC W-prep 2XY, and YAMAZEN on a disposal high flash column (silica gel). LC/ESI-MS, Liquid chromatography/electrospray ionization tandem mass spectrometry.

Synthesis of L687

2-Bromo-*N*-(2-ethoxyphenyl)acetamide (**8**)

To a solution of phenetidine (**7**, 1.372 g, 10 mmol) and triethylamine (1.33 mL, 10 mmol) in CH_2_Cl_2_ (10 mL) was slowly added bromoacetyl bromide (870 µL, 10 mmol) at 0 ^o^C. The mixture was stirred for 3 h at 0 °C, allowed to stand at room temperature, quenched with brine, and extracted with ethyl acetate. The organic layer was washed with sat. NaHCO_3_, dried over Na_2_SO_4_, and concentrated to give 2.5866 g of the title compound as a solid in a quantitative yield.

^１^H NMR (CDCl_３_, 400 MHz) δ8.96 (br, 1 H), 8.31 (dd, 1 H, J = 2.0, 8.0 Hz), 7.07 (dt, 1 H, J = 2.0, 8.0 Hz), 6.97 (dt, 1 H, J = 1.2, 8.0 Hz), 6.87 (dd, 1 H, J = 1.2, 8.0 Hz), 4.12 (q, 2 H, J = 6.8 Hz), 4.05 (s, 2 H), 1.48 (t, 3 H, J = 6.8 Hz), LC-MS: r.t. 2.58 min., m/z 258.0, 260.0 (M^+^ + 1).

*tert*-Butyl 4-(2-chloro-4-(methoxycarbonyl)phenyl)piperazine-1-carboxylate (**3**)

A mixture of **2** (5.04 g, 26.8 mmol), **1** (5.4337 g, 26.8 mmol) and DBU (4800 µL, 32.16 mmol) in DMSO (20 mL) was heated at 100 ^o^C for 10 h. The mixture was then diluted with sat. NH_4_Cl, and extracted with ethyl acetate. The organic layer was washed with brine, dried with Na_2_SO_4_ and concentrated. The residue was purified by preparative silica gel column chromatography with hexane/ethyl acetate to give 6.71 g of the title compound (Rf = 0.38, 5 : 1 hexane/ethyl acetate) in 68% yield.

^１^H NMR (CDCl_３_, 400 MHz) δ8.02 (d, 1 H, J = 2 Hz), 7.87 (dd, 1 H, J = 2.0, 8.0 Hz), 6.99 (d, 1 H, J = 8.0 Hz), 3.88 (s, 3 H), 3.54 - 3.64 (br, 4 H), 3.02 - 3.12 (br, 4H), 1.48 (s, 9 H),LC/MS R.T. = 3.34 min, m/z 355.1 (M^+^ + 1).

4-(4-(*tert*-Butoxycarbonyl)piperazin-1-yl)-3-chlorobenzoic acid (**4**)

A mixture of **3** (3.4 g, 9.22 mmol), 1.1 N NaOH (20 mL, 22 mmol), THF (20 mL) and MeOH (20 mL) was stirred at r.t. overnight. The mixture was neutralized with 1 N HCl and extracted with ethyl acetate. The organic layer was then washed with brine, dried over Na_2_SO_4_ and concentrated to give the product (2.86 g) in 91% yield.

^１^H NMR (CDCl_３_, 400 MHz) δ8.10 (d, 1 H, J = 2.0 Hz), 7.95 (dd, 1 H, J = 2.0, 8.8 Hz), 7.03 (d, 1 H, J = 8.8 Hz), 3.54 - 3.69 (br, 4 H), 3.02 - 3.19 (br, 4H), 1.49 (s, 9 H),LC/MS R.T. = 2.84 min, m/z 341.1 (M^+^ + 1).

*tert*-Butyl 4-(2-chloro-4-(piperidine-1-carbonyl)phenyl)piperazine-1-carboxylate (**5**)

A mixture of piperidine (891 µL, 9 mmol), **4** (2045 mg, 6 mmol), HATU (2.4 g, 6.3 mmol), and diisopropylethylamine (1254 µL, 7.2 mmol) in DMF (5 mL) was stirred overnight at room temperature. The mixture was extracted with ethyl acetate, and the organic layer was washed with sat. NaHCO_3_ and then sat. NH_4_Cl, dried over Na_2_SO_4_ and concentrated. The residue was purified by preparative silica gel column chromatography with hexane/ethyl acetate to give the desired product (2.3089 g, Rf = 0.43, 1 : 1 = hexane/ethyl acetate)) in 94% yield.

^１^H NMR (CDCl_３_, 400 MHz) δ7.42 (d, 1 H, J = 2 Hz), 7.29 (dd, 1 H, J = 2.0, 8.4 Hz), 6.99 (d, 1 H, J = 8.4 Hz), 3.3 - 3.8 (br, 8 H), 2.93 - 3.13 (br, 4H), 1.53 – 1.8 (br, 6 H) 1.48 (s, 9 H), LC/MS: r.t. 3.03 min, m/z 408.0 (M^+^ + 1).

(3-Chloro-4-(piperazin-1-yl)phenyl)(piperidin-1-yl)methanone dihydrochloride (**6**)

To a solution of 1-(*tert*-butoxycarbonyl)piperidine (2.30 g, 5.64 mmol) in ethyl acetate (10 mL), 4 M HCl/1, 4-dioxane (10 mL, 40 mmol) was added at room temperature, and the mixture was stirred overnight. The mixture was concentrated using a rotary evaporator and the resulting residual solid was suspended in ethyl acetate. The solid was collected by filtration, washed with ethyl acetate, and dried in vacuo to obtain the desired product (2.0641 g, 5.22 mmol) in 92% yield.

^１^H NMR (DMSO-d_6_, 400 MHz) δ8.81 (br, 2 H), 7.41 (d, 1 H, J = 2 Hz), 7.30 (dd, 1 H, J = 2.0, 8.4 Hz), 7.20 (d, 1 H, J = 8.4 Hz), 3.14 - 3.34 (br, 12 H), 1.35 – 1.64 (br, 6 H) 1.48, LC/MS: r.t. 1.52 min, m/z 308.2 (M^+^ + 1).

2-(4-(2-Chloro-4-(piperidine-1-carbonyl)phenyl)piperazin-1-yl)-*N*-(2-ethoxyphenyl)acetamide (**9**, L687)

Bromide **8** (516 mg, 2 mmol) and **6** (2 mmol) were suspended in DMF (5 mL) and DBU (3 mL, 20 mmol) was added to the mixture at room temperature. The resulting mixture was stirred for 6 h at 60 °C and then quenched with sat. NH_4_Cl, and extracted with ethyl acetate. The organic layer was washed by sat, NaHCO_3_, dried over Na_2_SO_4_ and concentrated. The residue was purified by preparative silica gel column chromatography with hexane/ethyl acetate to give 356 mg of the title compound in 46% yield, together with the crude product (75 mg).

^１^H NMR (CDCl_３_, 400 MHz) δ9.87 (bs, 1 H), 8.44 (dd, 1 H, J = 1.6, 8.0 Hz), 7.53 (d, 1 H, J = 2.0 Hz), 7.29 (dd, 1 H, J = 2.0, 8.4 Hz), 7.03 (dt, 1 H, J = 2.0, 7.6 Hz), 7.03 (d, 1 H, J = 8.0 Hz), 6.96 (dt, 1 H, J = 1.2, 7.6 Hz), 6.87 (dd, 1 H. J = 1.6, 8.0 Hz), 4.11 (q, 2 H, J = 6.8 Hz), 3.52 – 3.75 (br, 2 H), 3.31 – 3.52 (br, 2 H), 3.25 (s, 2 H), 3.12 – 3.22 (br, 4H), 2.80 – 2.88 (brt, 4 H), 1.65 – 1.73 (br, 4 H), 1.54 – 1.65 (br, 2 H), 1.51 (t, 3 H, J = 6.8 Hz) LC-MS: r.t. 2.31 min., m/z 485.1 (M^+^ + 1).

**Supplementary Figure 2.**

**
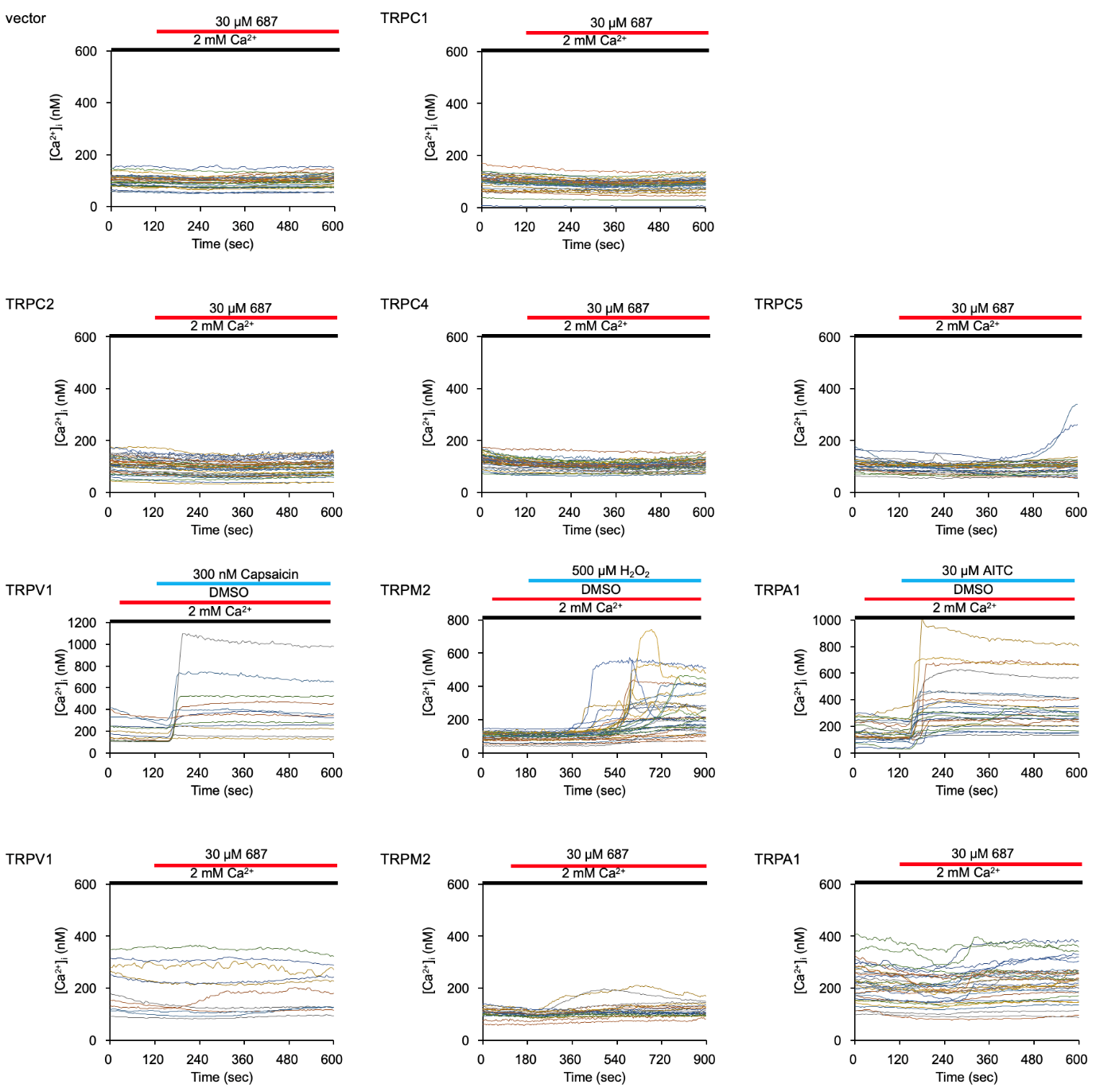
**

Intracellular Ca^2+^ influx analysis in each overexpressed TRP channel. Analysis of Ca^2+^ influx into A549 cells in the presence of 2 mM Ca^2+^. Ca^2+^ influx was recorded after adding L687 (30 µM). AITC, allyl isothiocyanate; TRP, transient receptor potential.

**Supplementary Figure 3.**


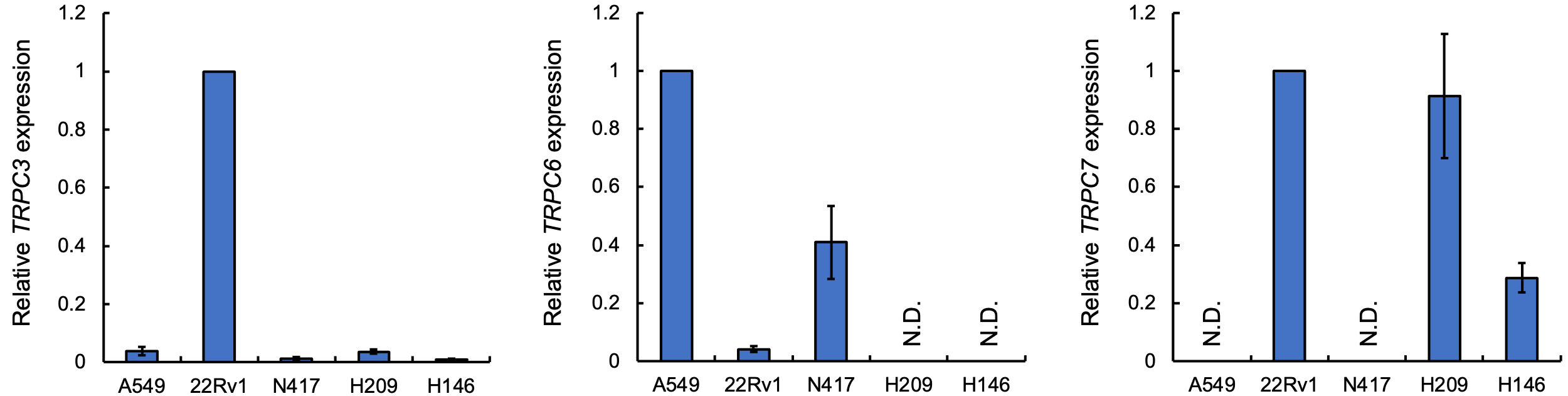


RT-qPCR analysis of TRPC3/C6/C7 expression in various cell lines. *TRPC3/6/7* expression was analysed in cell lysates using RT-qPCR. Relative expression was compared with the highest expression of 1. N.D., not detected; RT-qPCR, reverse transcription-quantitative PCR; TRPC, transient receptor potential canonical.

**Supplementary Figure 4.**


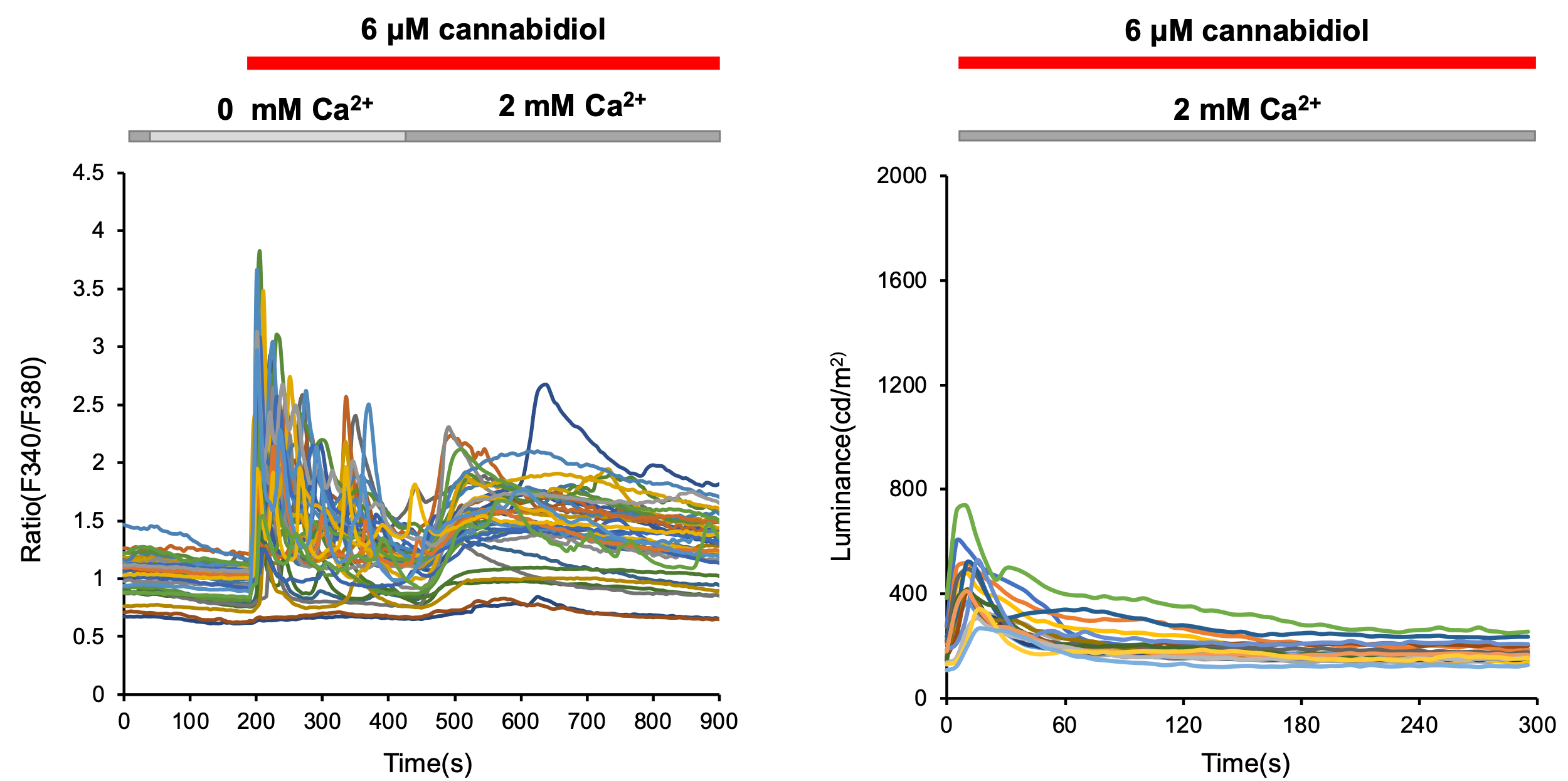


Ca^2+^ imaging of TRPC6 channel activation by cannabidiol (CBD) in HEK293 cells overexpressing TRPC6. Analysis of the intracellular Ca^2+^ influx following the addition of 6 μM CBD in HEK293 cells overexpressing TRPC6 in the presence of 2 mM Ca^2+^ in the medium. Ca^2+^ influx was recorded following the addition of CBD. TRPC, transient receptor potential canonical.

**Supplementary Figure 5.**


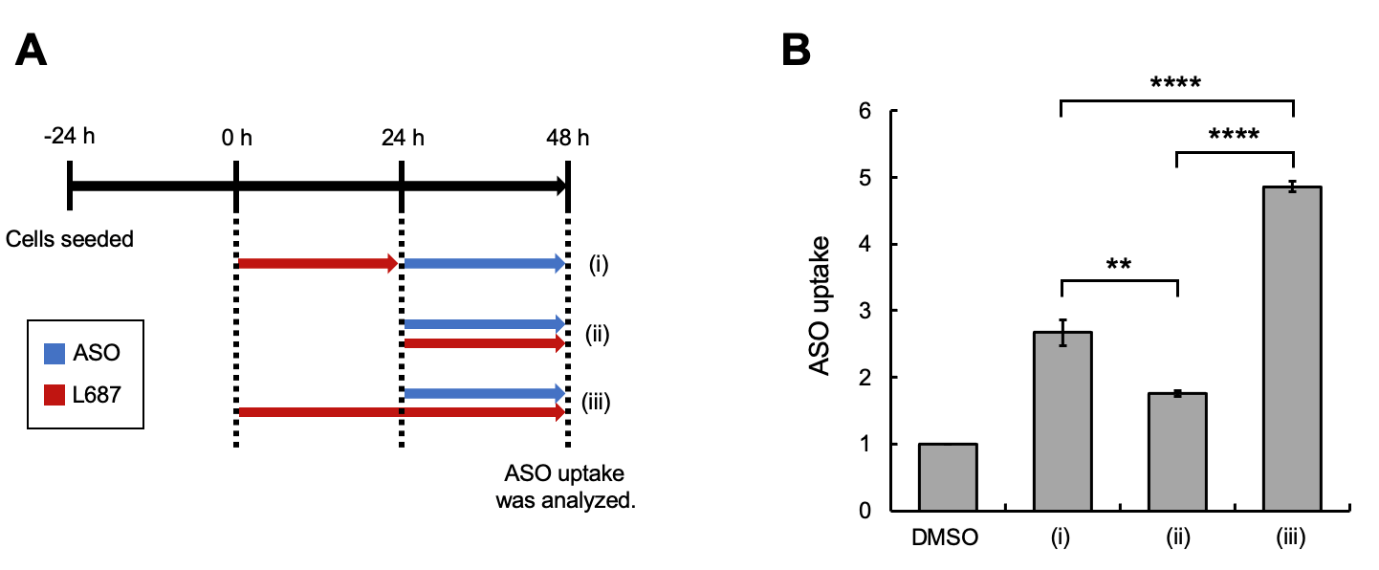


Effect of L687 addition at different time points on ASO uptake. (**A**) (i) L687 was added for 24 h, and the medium was replaced with ASO without L687. (ii) ASO with L687 was added to the medium for 24 h. (iii) L687 was added to the medium, and ASO was added after 24 h. The cells were then cultured for 24 h. (**B**) Relative ASO uptake was analysed in A549 cells. Alexa647-AmNA#26 (10 nM) with 30 µM L687 was added to the medium under conditions shown in (**A**), and intracellular fluorescence intensity was analysed by flow cytometry after 24 h. The relative MFI is shown, compared with that of the medium containing DMSO. Data are presented as mean ± standard error of the mean (SEM) of six independent experiments (n=6). Statistical significance was compared with the values for DMSO using Tukey’s test. ***p*<0.01, *****p*<0.0001. ASO, antisense oligonucleotide; DMSO, dimethyl sulfoxide; MFI, mean fluorescence intensity.

**Supplementary Figure 6.**


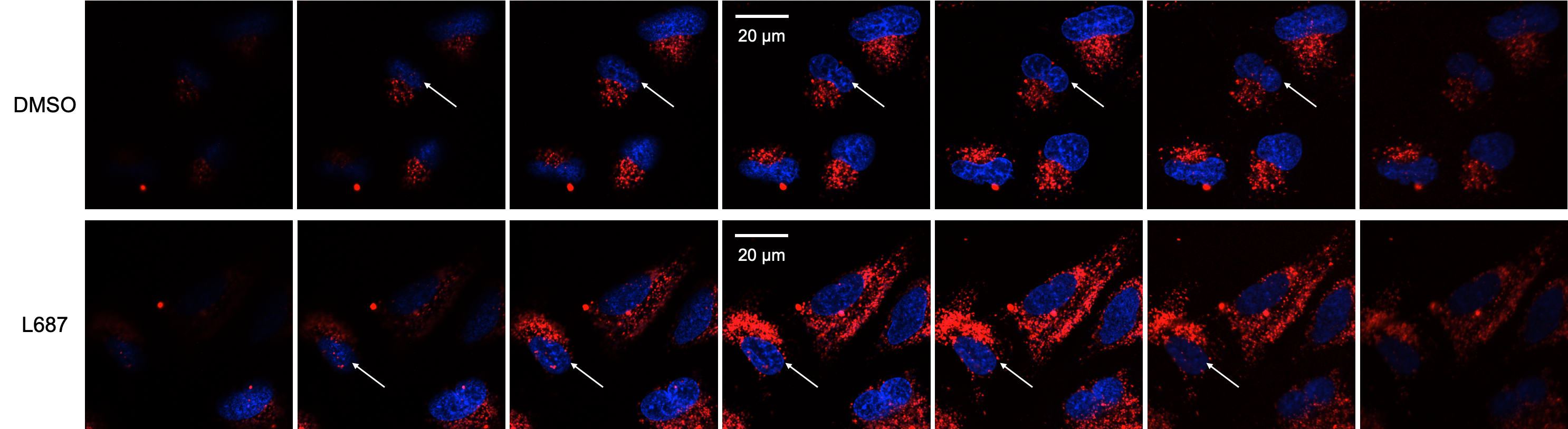


Confocal Z-stack images of ASO incorporated into A549 cells. Alexa647-AmNA#26 (100 nM) and L687 (30 µM) were added to the medium, and Hoechst staining was performed after 48 h. Z-stack images were acquired from the bottom to the top of the cells. Optical sections were gathered in 1 µm steps perpendicular to the z-axis (microscope optical axis). ASO, antisense oligonucleotide

**Supplementary Figure 7.**


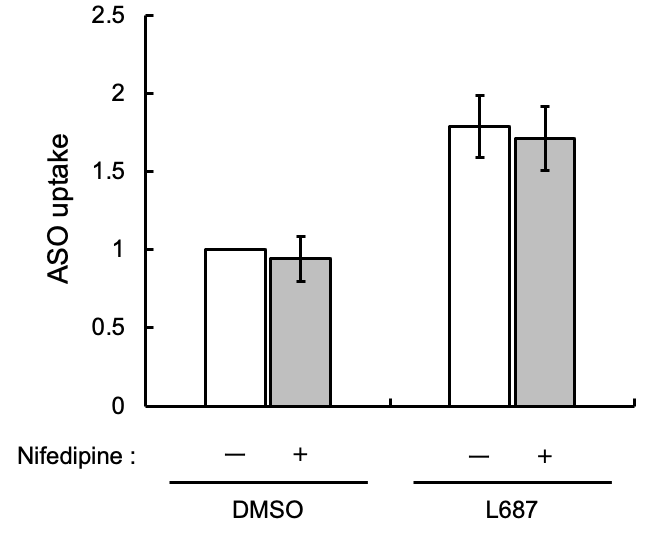


Analysis of ASO uptake by incubating A549 cells with L-type calcium channel blocker, nifedipine. ASO and L687 were added to the medium with or without nifedipine (10 µM). After 24 h, intracellular fluorescence intensities were analysed by flow cytometry. ASO, antisense oligonucleotide; DMSO, dimethyl sulfoxide; MFI, mean fluorescence intensity.

**Supplementary Figure 8.**


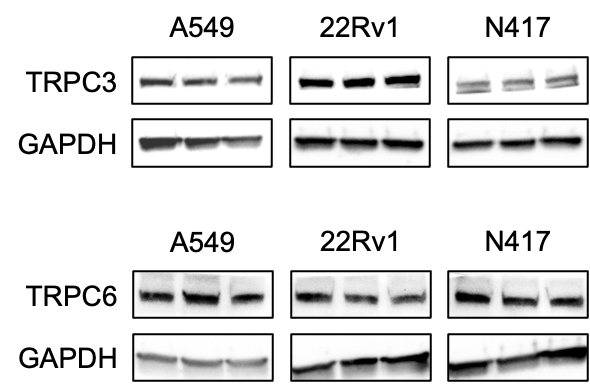


Western blot analysis of TRPC3/C6 expression in various cell lines. Western blots of three independent experiments in each cells were depicted. TRPC, transient receptor potential canonical.
